# Self-assembly of chiral fluorescent nanoparticles based on water-soluble L-tryptophan derivatives of *p-tert-*butylthiacalix[4]arene

**DOI:** 10.3762/bjnano.8.184

**Published:** 2017-09-04

**Authors:** Pavel L Padnya, Irina A Khripunova, Olga A Mostovaya, Timur A Mukhametzyanov, Vladimir G Evtugyn, Vyacheslav V Vorobev, Yuri N Osin, Ivan I Stoikov

**Affiliations:** 1 Kazan Federal University, 420008 Kremlevskaya, 18, Kazan, Russian Federation; 2 Peoples Friendship University of Russia (RUDN University), 117198 Miklukho-Maklaya St., 6, Moscow, Russian Federation

**Keywords:** fluorescence, nanoparticles, self-assembly, thiacalixarene, tryptophan

## Abstract

New water-soluble tetra-substituted derivatives of *p-tert*-butylthiacalix[4]arene containing fragments of L-tryptophan in *cone* and 1,3-*alternate* conformations were obtained. It was shown that the resulting compounds form stable, positively charged aggregates of 86–134 nm in diameter in water at a concentration of 1 × 10^−4^ M as confirmed by dynamic light scattering, scanning electron microscopy and transmission electron microscopy. It was established that these aggregates are fluorescently active and chiral. A distinctive feature of the compounds is the pronounced dependence of their spectral (emission and chiroptical) properties on the polarity of the solvent and the length of the linker between the macrocyclic and fluorophore parts of the molecule.

## Introduction

Recently, fluorescently active compounds have become a high demand in nanotechnology, biotechnology and medicinal chemistry [1–2]. Nanoparticles that consist of covalently or non-covalently bound fluorescent compounds are widely used due to attractive physical and chemical properties [3–4]. Water-soluble stable fluorescent nanoparticles open up new opportunities for the design of particles that can be traced throughout the body, for example, for the delivery of therapeutic agents [5], synthetic vectors for gene therapy [6] and contrast agents for magnetic resonance imaging [7–8]. Similarly, supramolecular particles can consist of various organic or inorganic components. However, most of the fluorescent water-soluble nanoparticles described in the literature are metal-based (silver, gold, copper, etc.) [9–12]. Noncovalent self-assembly is a promising approach for creating fluorescent organic nanoparticles. Special attention is focused on the use of building blocks for the preparation of the nanoparticles from the macrocyclic compounds such as cyclodextrins, cucurbit[*n*]uriles, calix[*n*]arenes, pillar[*n*]arenes and others [13–34]. The enormous interest in the use of these compounds is explained by their complexing properties, which make it possible to obtain nanomaterials with practical useful properties.

We have previously obtained water-soluble supramolecular nanomaterials based on *p-tert-*butylthiacalix[4]arene in *cone* conformation capable of recognizing peptides [35]. Similar compounds in *cone* and 1,3-*alternate* conformations containing peptide fragments as substituents were able to form complexes with DNA [36]. In both cases, solubility in water was achieved by introducing positively charged ammonium groups into the structure of macrocycles.

**Figure 1:**
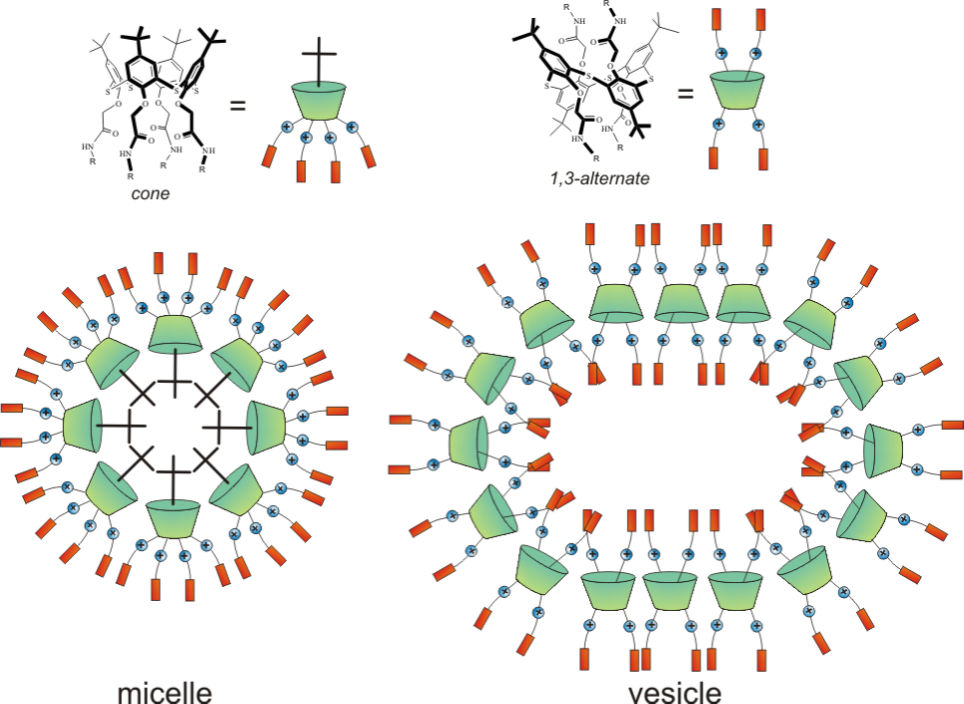
Possible paths of the formation of supramolecular associates.

In this paper, an approach to obtain the water-soluble fluorescent chiral nanoparticles based on derivatives of tetra-substituted *p-tert*-butylthiacalix[4]arene containing L-tryptophan fragments at the lower rim in *cone* and 1,3-*alternate* conformations was developed. It is possible to form various types of self-assemblies depending on the conformation of the thiacalixarenes (*cone* or 1,3-*alternate*). According to the literature data [37–38], the spatial arrangement of the substituents against the macrocyclic platform was a fundamental matter for the structure of the resulting supramolecular associates (Figure 1).

Also the influence of the structure and conformation of *p-tert*-butylthiacalix[4]arenes on their ability to self-assemble into different types of associates as well as the chiroptical and fluorescent properties of the compounds are studied.

## Results and Discussion

In our group [26–27,35–36] a step-by-step synthesis of tetra-substituted water-soluble ammonium derivatives of *p-tert-*butylthiacalix[4]arene was developed. The approach consists of the introduction of an amino group in the substituents at the lower rim of *p-tert*-butylthiacalix[4]arene with the subsequent interaction of the resulting amine with various alkylating reagents by the Menshutkin reaction.

In order to obtain quaternary ammonium salts, the macrocycles **4**–**7**, containing both secondary amide and tertiary amino groups at the lower rim in *cone* and 1,3-*alternate* conformations, were chosen as precursors. The alkylating agent containing a fluorescent active fragment of L-tryptophan was suggested to be used as a second reagent for the Menshutkin reaction.

**Scheme 1:**
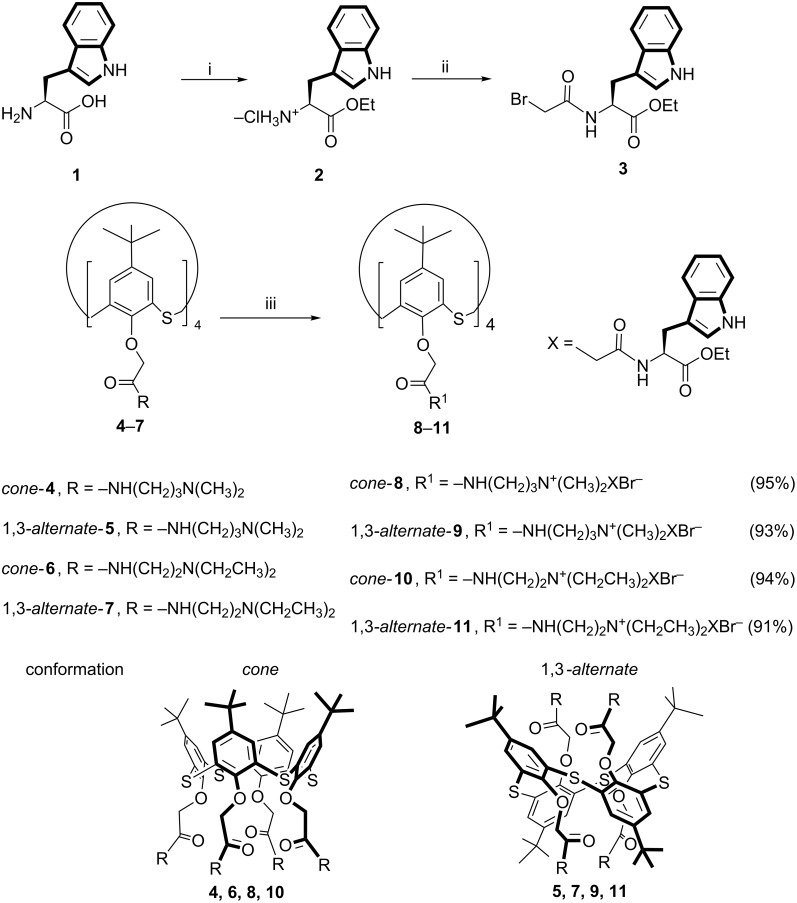
Reagents and conditions: (i) SOCl_2_, C_2_H_5_OH, (ii) BrCH_2_COBr, NaHCO_3_, C_6_H_6_/H_2_O, (iii) **3**, CH_3_CN, reflux.

*N*-bromoacetyl-L-tryptophan ethyl ester **3** was obtained by the acylation of L-tryptophan ethyl ester hydrochloride **2** with bromoacetyl bromide carried out in benzene at room temperature according to the literature procedure [39] (Scheme 1).

The alkylating reagent **3** was chosen owing to the presence of aromatic fragments in the structure of the chiral amino acid. The ammonium derivatives of thiacalix[4]arene can form self-associates not only due to hydrogen bonds, but also due to π–π-stacking and the hydrophobic effects of the chiral amino acid with aromatic fragments [40]. The presence of amide fragments in the structure of the resulting macrocycles also contributes to the formation of aggregates via self-association resulting from the formation of hydrogen bonds. Further, the interaction of the alkylating reagent **3** with aminothiacalixarenes **4**–**7** containing methyl and ethyl fragments at the tertiary nitrogen atom in the *cone* and 1,3-*alternate* conformations was studied (Scheme 1). The reaction was carried out in acetonitrile under 8 h reflux. In the case of the initial tetraamines **4** and **5**, the ammonium derivatives **8** and **9** were obtained with high yields. The dimethyl-substituted tertiary amino groups at the lower rim in thiacalix[4]arenes **4** and **5** were found to be more reactive than the analogous diethyl-substituted amino groups in compounds **6** and **7**. This is probably caused by the different steric hindrance at the tertiary amino groups. Increasing the reaction time from 8 to 40 h afforded compounds **10** and **11** in high yields (Scheme 1). It should be noted that despite of the bulk lipophilic indole fragment in the structure of L-tryptophan, the resulting compounds **8**–**11** are water soluble. The structure and composition of the synthesized compounds **8**–**11** were determined by ^1^H and ^13^C NMR, IR spectroscopy, ESI mass spectrometry and elemental analysis.

The method of dynamic light scattering (DLS) is widely used to study colloidal particles, especially solutions of macromolecules and molecular ensembles [41]. The solutions of thiacalix[4]arenes **8**–**11** at different concentrations (1 × 10^−4^–1 × 10^−6^ М) were studied in water (Table 1).

**Table 1:** Diameter and polydispersity index (PDI) of macrocycles **8**–**11** at different concentrations in water.

Thiacalix[4]arene concentration	Particle diameter (nm), PDI			
	**8**	**9**	**10**	**11**

1 × 10^−4^ М	120 ± 12 (0.26)	131 ± 3 (0.30)	134 ± 3 (0.21)	86 ± 1 (0.22)
1 × 10^−5^ М	208 ± 27 (0.39)	184 ± 15 (0.34)	236 ± 10 (0.34)	162 ± 18 (0.34)
1 × 10^−6^ М	323 ± 37 (0.48)	292 ± 17 (0.37)	258 ± 11 (0.34)	212 ± 23 (0.36)

Regardless the macrocycle conformation, a monotonous increase in the diameter of the macrocycle aggregates **8**–**11** is observed with concentration decreasing from 1 × 10^−4^ to 1 × 10^−6^ М. The polydispersity value (PDI) increased simultaneously (Table 1).

A similar trend has been described for other water-soluble thiacalix[4]arene derivatives containing glycine fragments at the lower rim [36]. This is probably due to the changing form of the self-associates with the concentration of macrocycles. For the macrocycles **8**–**11**, the most stable monodisperse systems are formed at the concentration of 1 × 10^−4^ М (Table 1, Supporting Information File 1, Figures S17–S20). It was shown that with time (within 7 days) the values of polydispersity and hydrodynamic diameter of particles in solutions of the thiacalixarenes **8**–**11** did not change. This also indicates the high stability of these systems. It should be noted that the replacement of water with methanol does not lead to the formation of aggregates of the compounds **8**–**11**.

The zeta potential is another measure of the stability of colloidal systems. The zeta potential characterizes the degree and nature of the interaction between the particles of the disperse system: the larger the electrokinetic potential, the more stable the colloidal system. A low zeta potential determines the tendency of the particles of a colloidal solution to coagulate and flocculate. It has been experimentally established [42] that the critical value of the zeta potential corresponding to the stability threshold of a colloidal system is 30 mV.

The zeta potential of the thiacalix[4]arene solutions **8**–**11** was determined at a concentration of 1 × 10^−4^ М in water (Table 2, Supporting Information File 1, Figure S21–S24). Large zeta potential values (more than 30 mV) also confirm the high stability of the aggregates of these compounds. For the associates of the compound **11**, which have the smallest diameter (86 ± 1 nm), the value of the zeta potential (+40 mV) is also the lowest. It can be assumed that the smaller nanoparticles of compound **11** have a smaller diffuse layer, and therefore have the lowest zeta potential value in the series of the compounds **8**–**11**.

**Table 2:** The zeta potential of solutions of thiacalix[4]arenes **8**–**11** at a concentration of 1 × 10^−4^ М in water.

Zeta potential, mV			
**8**	**9**	**10**	**11**

+67 ± 1	+65 ± 1	+65 ± 1	+40 ± 1

**Figure 2:**
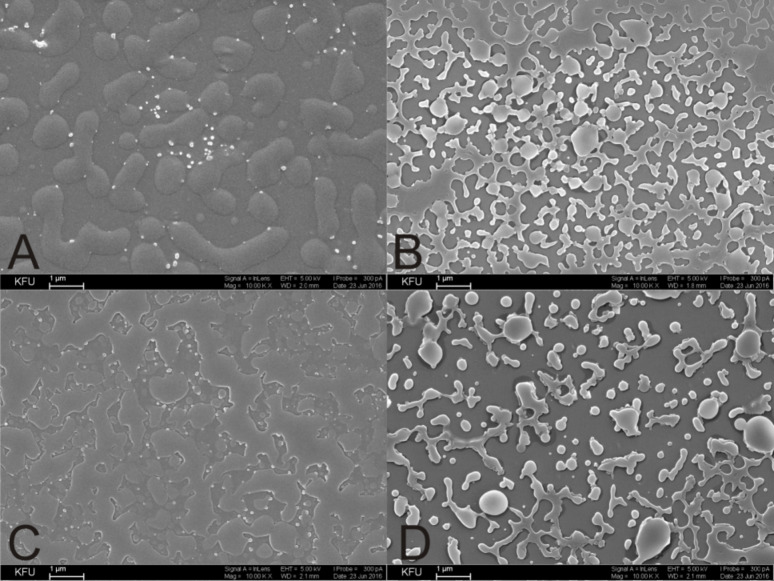
SEM images of self-associates based on thiacalix[4]arene: А) compound **8**, B) compound **9**, C) compound **10**, D) compound **11** in water (1 × 10^−4^ М). Scale bar 1 µm.

The nanoscale aggregates formed by the *p-tert-*butylthiacalix[4]arenes **8**–**11** containing amide, quaternary ammonium and amino acid fragments in *cone* and 1,3*-alternate* conformation were also investigated by transmission electron microscopy (TEM) and scanning electron microscopy (SEM) (Figure 2, Supporting Information File 1, Figures S25–S28). Electron microscopy can uniquely determine the size and shape of aggregates. The existence of spherical nanoparticles with diameters very close to those determined by DLS in the case of the derivatives **8** and **10** in *cone* conformation was confirmed. In the case of the derivatives **9** and **11** in 1,3*-alternate* conformation, both individual nanometer-scale particles and large aggregates as well as branched structures consisting of these particles were detected. Apparently, micellar-type structures are formed in aqueous solutions of the amphiphilic macrocycles **8** and **10** in *cone* conformation. In the case of the thiacalixarenes **9** and **11** in 1,3-*altenate* conformation, branched structures were observed owing to the possibility of the formation of additional hydrogen bonds between the associates due to the arrangement of substituents that are on both sides of the macrocyclic ring. Good agreement was observed between the DLS data and the microscopy measurements in the case of the compounds **8** and **10** in *cone* conformation. This is due to the structure of the macrocycles, which formed spherical charged particles (micelles) with inwardly directed hydrophobic parts (thiacalixarene) and outwardly oriented hydrophilic positively charged fragments in the solution (Figure 1). The charge on the micelles prevents them from aggregating when we concentrate the solutions for preparation of the SEM experiment. This leads to the preservation of individual particles of similar size to those that exist in solution. The conformers of the 1,3*-alternate* (the compounds **9** and **11**) have a fundamentally different structure with hydrophilic fragments located on both sides of the macrocyclic system (Figure 1). As a result, they form extended vesicles with a hydrophobic part disposed between two layers of polar charged fragments. The concentration of the solutions leads to the coalescence of the particles by hydrophobic fragments, and the particles are hence considerably enlarged. This results in aggregates that are much larger than those in solution which are visible in the SEM images.

Tryptophan is an amino acid with significant fluorescence [43]. The ability to fluoresce is due to the presence of the indole groups in the structure of the compounds **8**–**11**. Currently, fluorescently active, water-insoluble tryptophan derivatives of the calix[4]arene, capable of selective binding of fluoride anions, some metal cations and amino alcohols, were obtained [44–48]. Thus, the functionalization of the macrocyclic thiacalix[4]arene platform with tryptophan fragments can be useful in creation of new sensory materials. The great conformational ability of this macrocycle in combination with the use of templating synthesis methods makes it possible to obtain a wide range of the structures with binding centers preorganized and rigidly fixed in space. Besides, the preparation of water-soluble fluorescent compounds is a perspective field, which opens the possibility for detecting various water-soluble biologically important substrates and for analyzing biological fluids.

**Figure 3:**
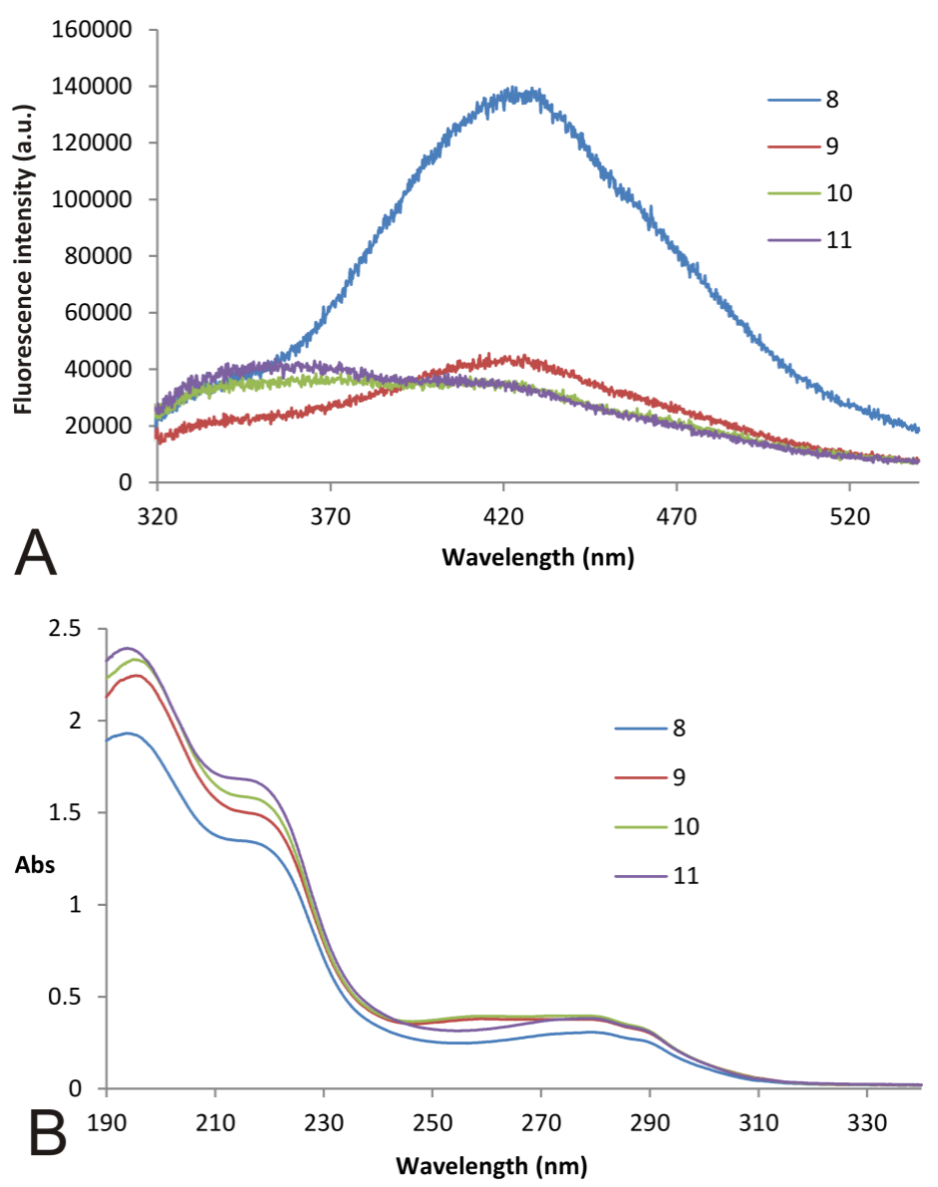
A) Fluorescence spectra of compounds **8**–**11** in water (1 × 10^−5^ М), B) UV spectra of aqueous solutions of thiacalix[4]arenes **8**–**11** (1 × 10^−5^ М).

Tryptophan has a maximum emission in water at 348 nm. The position of the maximum strongly depends on the polarity of the solvent [43]. The emission maximum of the compounds **8** and **9** in water was observed at 415 nm. It was shifted to the red region in comparison with the compounds **10** and **11** (349 nm) and the initial ester **2** (Figure 3A, Supporting Information File 1, Figure S29). The dependence of fluorescence on the conformation of macrocycles was virtually absent for both pairs of the compounds **8**, **9** and **10**, **11**. The intensity of the emission spectrum at the emission maximum is more than three times higher for the compound **8** in *cone* conformation than for the compound **9** in the 1,3*-alternate* conformation (Figure 3A). Meanwhile, the intensity of emission of both conformations is about the same for the compounds **10** and **11** containing *N*,*N*-diethyl fragments. To explain all these facts, we recorded the absorption spectra of the compounds **8–11** and the ester **2**. The spectra of all these compounds are identical: there is an absorption band of low intensity corresponding to the π→π* transition of the indole fragment in the longwave region [49] and it is located at 280 nm for all synthesized compounds (Figure 3B). Such a large Stokes shift for the methyl compounds **8** and **9** (135 nm) is attributed to the formation of excimers. It is known that indole fragments are capable of π–π-stacking, which makes the formation of excimers possible [50–51].

We propose that compounds **10** and **11**, with ethyl substituents at the ammonium nitrogen atom, are not capable of forming excimers due to the repulsion of positively charged ammonium groups. The short length of the chain between the macrocyclic and tryptophan parts does not allow adjacent tryptophan fragments to come close enough to one another for excimer formation. This is demonstarted by a slight Stokes shift (69 nm) for these compounds, which refers to the fluorescence of the free monomer. The elongation of the hydrocarbon chain per methylene moiety for methyl compounds **8** and **9** already removes these steric obstacles and the formation of excimers becomes possible. At the same time, the difficulty in excimer formation in the case of compounds **10** and **11** may be caused by the presence of bulky ethyl fragments. However, in our opinion, this is not so important, because it was shown [47] that there is a significant difference in emission wavelengths for the two calixarenes with bulk Boc groups in the tryptophan nitrogen atom, which differ only in the linker length between the macrocyclic and tryptophan parts. Therefore, we assert that only the length of the linker exerts an influence on the emission spectra.

There is no effect of the conformation on the position of the emission maxima and on the shape of the spectra of the studied compounds, which is characteristic for thiacalixarene derivatives [52–53]. This is probably due to the fact that the substituents are long and removed from the macrocyclic fragment, and the shielding of tert-butyl groups by fluorophores is not observed. As a result, the spectral pattern does not depend on the conformation in the pairs of compounds **8**, **9** and **10**, **11**.

**Figure 4:**
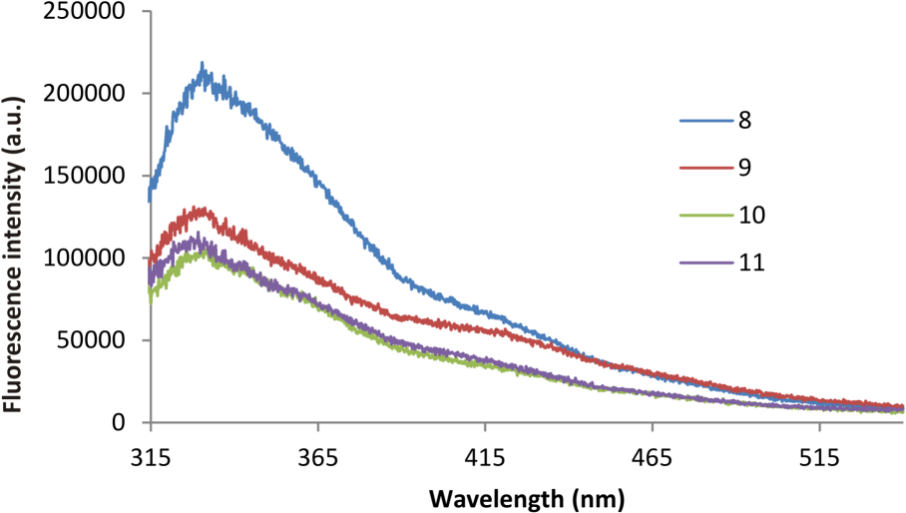
Fluorescence spectra of compounds **8**–**11** in methanol (1 × 10^−5^ М).

For a more detailed study of fluorescent properties, we recorded UV and fluorescence spectra of compounds **2** and **8**–**11** in methanol (Figure 4, Supporting Information File 1, Figure S29). It turned out that the emission maxima are close for all compounds in methanol, which indicates the absence of excimer formation. Perhaps this is due to the fact that in water, as a more polar solvent (dielectric constant 78.36) compared with methanol (32.66) [54], a complex with water is formed, leading to ionization of the indole [55–57]. As a result, the interaction between the ionized and neutral indole fragments in the macrocycle is enhanced, which leads to the formation of an excimer. Methanol, a less polar solvent, probably does not contribute to excimer formation.

It is known that circular dichroism (CD) spectroscopy is widely used for the analysis of the secondary structure of proteins [58]. It is possible to assume exactly how the molecule of the linear polypeptide is packed in space by the form of the CD spectrum. It can be α-helix, antiparallel β-sheet, type I and II β-turn, irregular structure, etc. The presence of four fragments of the chiral amino acid L-tryptophan in the structure of the thiacalix[4]arenes **8**–**11** allows for the assumption of the presence of the Cotton signal in the CD spectra.

The compounds **8** and **9** are able to form excimers in water, so the influence of the solvent (water or methanol) on the CD spectra was studied. The CD spectra of the compounds **8**–**11** (5 × 10^−5^ М, concentration of the tryptophan fragments 2 × 10^−4^ М) and the L-tryptophan ethyl ester hydrochloride **2** (2 × 10^−4^ М) were recorded in water and methanol.

**Figure 5:**
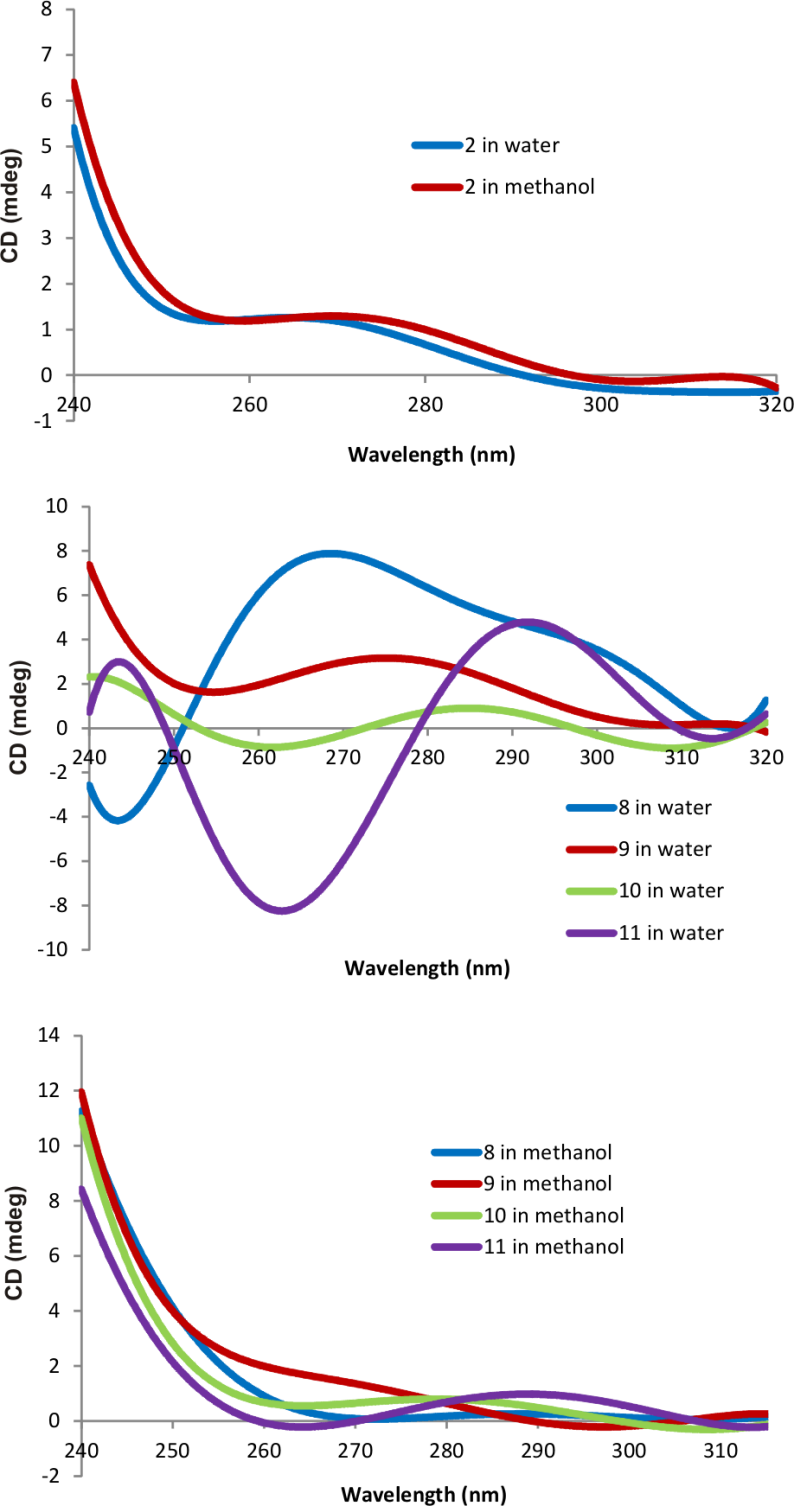
The CD spectra of the compounds **2** and **8**–**11** in water and methanol.

As expected, the CD spectra of the L-tryptophan ethyl ester hydrochloride **2** in water and methanol are almost identical. However, the CD spectra of the compounds **8**–**11** differ fundamentally in different solvents (Figure 5).

In methanol, the CD spectra of the compounds **8**–**11**, regardless of the conformation and substituents, are almost identical and similar to the CD spectrum of the L-tryptophan ethyl ester hydrochloride **2**. This is expected due to the absence of self-aggregation of the compounds studied in methanol, as established by DLS. There is a strong difference in the spectra of the CD pairs of the compounds **8**, **9** and **10**, **11** when methanol is substituted by water. In the CD spectra of the compounds **10** and **11** containing ammonium fragments with ethyl substituents, a negative Cotton effect was observed in the interval 250–280 nm and a positive Cotton effect in the interval 280–320 nm. The compounds **8** and **9** form excimers upon UV irradiation. In the case of the compounds **8** and **9**, a positive Cotton effect was found in the interval of 250–320 nm. But for the compound **8** a negative Cotton effect was also observed in the short-wavelength region of the spectrum (up to 250 nm). Thus, the set of data obtained by DLS, fluorescence and UV–visible spectroscopy, together with the negative Cotton effect results in the intervals 280–320 and 240–250 nm, indicate the formation of chiral nanoparticles in water. In analogy with proteins [58], these pairs of the compounds (**8**, **9** and **10**, **11**) form a different secondary structure in water, i.e., additional packing of tryptophan fragments in dimer formations is observed for the compounds **8** and **9** with methyl groups at the nitrogen atom, which leads to changes in the form of the CD spectrum. The optical activity of the systems was also characterized with anisotropy *g*-factors. The obtained compounds form nanoparticles in water with appreciable chiroptical activity with *g*-factors reaching 1.89 × 10^−4^ in the UV region of the spectrum (Supporting Information File 1, Figure S30).

## Conclusion

New water-soluble tetra-substituted lower-rim derivatives of *p-tert*-butylthiacalix[4]arene containing fragments of L-tryptophan in *cone* and 1,3*-alternate* conformations were obtained. Using the DLS method, it was shown that the resulting compounds formed stable positively charged aggregates of 86–134 nm in size in water at a concentration of 1 × 10^−4^ M. It was established by a number of optical methods that these aggregates were fluorescently active and chiral. The pronounced dependence of their spectral (emission and chiroptical) properties on the polarity of the solvent and the length of the linker between the macrocyclic and fluorophore parts of the molecule is a distinctive feature of the compounds obtained. It was shown that the elongation of the linker and the increase of the polarity of the solvent led to a significant increase of the Stokes shift due to excimer formation. It was established that changes in the CD spectra also confirmed the change in the structure of the nanoaggregates. The results can be useful in the design of fluorescently active compounds with predetermined emission parameters. It seems important in the future to extend the study on the D-amino acid derivatives in order to clarify the nature of the established chiroptical effects.

## Experimental

The ^1^H and ^13^C NMR spectra were recorded on a Bruker Avance 400 spectrometer (400.17 MHz for H-atoms) for 3–5% solutions in DMSO-*d*_6_. The residual solvent peaks were used as an internal standard. The IR spectra were recorded on Spectrum 400 (Perkin Elmer) IR spectrometer. Elemental analysis was performed on Perkin–Elmer 2400 Series II instruments. Mass spectra (ESI) were recorded on an AmaZonX mass spectrometer (Bruker Daltonik GmbH, Germany). The drying gas was nitrogen at 300 °С. The capillary voltage was 4.5 kV. The samples were dissolved in acetonitrile (concentration ≈10^−6^ g/mL). The melting points were determined using a Boetius Block apparatus. The purity of the compounds was monitored by melting points, boiling points, ^1^H NMR and thin layer chromatography (TLC) on 200 μm UV 254 silica gel plate using UV light. All experiments (NMR, UV–vis, CD spectroscopy and DLS) were performed at 298 K. The CD spectra were recorded on a Jasco-1500 spectrophotometer. The spectra were measured with scan rate of 50 nm/min, over a spectral range of 240–320 nm, with a slit width of 1 nm, sampling step of 1 nm and repeated for 3 scans.

In this work, the following reagents and solvents were used: acetonitrile (chemical pure), benzene (chemical pure), bromoacetyl bromide (chemical pure), L-tryptophan (Acros Organics), ethanol (chemical pure), thionyl chloride (chemical pure), magnesium sulfate (chemical pure), sodium bicarbonate (chemical pure).

5,11,17,23-Tetra-*tert*-butyl-25,26,27,28-tetrakis[(*N*-(3-(dimethylamino)propyl)carbamoylmethoxy]-2,8,14,20-tetrathiacalix[4]arene (*cone*-**4**) was synthesized according to the literature procedure [35].

5,11,17,23-Tetra-*tert*-butyl-25,26,27,28-tetrakis[(*N*-(3-(dimethylamino)propyl)carbamoylmethoxy]-2,8,14,20-tetrathiacalix[4]arene (1,3*-alternate*-**5**) was synthesized according to the literature procedure [36].

5,11,17,23-Tetra-*tert*-butyl-25,26,27,28-tetrakis[(*N*-(2-(diethylamino)ethyl)carbamoylmethoxy]-2,8,14,20-tetrathiacalix[4]arene (*cone*-**6**) was synthesized according to the literature procedure [35].

5,11,17,23-Tetra-*tert*-butyl-25,26,27,28-tetrakis[(*N*-(2-(diethylamino)ethyl)carbamoylmethoxy]-2,8,14,20-tetrathiacalix[4]arene (1,3*-alternate*-**7**) was synthesized according to the literature procedure [36].

**Procedure for the synthesis of L-tryptophan ethyl ester hydrochloride 2** [39]**:** In a 500 mL round bottom flask equipped with a magnetic stirrer L-tryptophan **1** (10.00 g, 49 × 10^−3^ mol) was dissolved in 150 mL ethanol. Thionyl chloride (98 × 10^−3^ mol) was added dropwise. The reaction mixture was stirred for 30 min at room temperature. Then the reaction mixture was refluxed for 4 h. The resulting product was filtered. The precipitate was washed with ethanol and dried under reduced pressure over phosphorus pentoxide.

L-tryptophan ethyl ester hydrochloride **2**: ^1^H NMR (400 MHz, 298 K, DMSO-*d*_6_) δ 1.08 (t, ^3^*J*_HH_ = 7.1 Hz, 3H, C*H*_3_CH_2_O–), 3.22–3.30 (m, 2H, CHC*H*_2Trp_), 4.07 (q, ^3^*J*_HH_ = 7.1 Hz, 2H, CH_3_C*H*_2_O–), 4.19 (m, 1H, NНC*H*СО), 6.99–7.53 (m, 5H, ArH_Trp_), 8.55 (s, 1Н, NH_3_^+^CH), 11.09 (s, 1Н, NH_Trp_).

**Procedure for the synthesis of *N*-bromoacetyl-L-tryptophan ethyl ester 3** [39]**:** In a 500 mL round bottom flask equipped with a magnetic stirrer and two dropping funnels at room temperature L-tryptophan ethyl ester hydrochloride **2** (36 × 10^−3^ mol) was added to mixture of 50 mL of a 10% aqueous solution of sodium bicarbonate and 50 mL of benzene. Then 50 mL of a 10% aqueous solution of sodium bicarbonate and 4 mL (36 × 10^−3^ mol) of a solution of bromoacetyl bromide in 50 mL of benzene were simultaneously added dropwise. The mixture was stirred for 5 h while maintaining a neutral pH. Then glacial acetic acid was added to pH 6.5. The organic phase was separated, dried over anhydrous magnesium sulfate, after which the benzene was removed under reduced pressure.

*N*-bromoacetyl-L-tryptophan ethyl ester **3**: ^1^H NMR (400 MHz, 298 K, DMSO-*d*_6_) δ 1.08 (t, ^3^*J*_HH_ = 7.1 Hz, 3H, C*H*_3_CH_2_O–), 3.04–3.18 (m, 2H, CHC*H*_2Trp_), 3.89 (s, 2H, BrC*H*_2_CO), 4.03 (q, ^3^*J*_HH_ = 7.1 Hz, 2H, CH_3_C*H*_2_O–), 4.50 (m, 1H, NНC*H*СО), 6.97–7.49 (m, 5H, ArH_Trp_), 8.73 (d, ^3^*J*_HH_ =7.5 Hz, 1Н, СОN*H*CH), 10.89 (s, 1Н, NH_Trp_).

**General procedure for the synthesis of the compounds 8**–**11:** In a round bottom flask equipped with a magnetic stirrer and a reflux condenser, the compound **4**–**7** (0.20 g, 0.15 × 10^−3^ mol) was dissolved in 10 mL acetonitrile. *N*-Bromoacetyl-L-tryptophan ethyl ester **3** (0.60 × 10^−3^ mol) was added. The reaction mixture was refluxed for 40 h. The solvent was removed under reduced pressure. The precipitate was washed with water and dried under reduced pressure over phosphorus pentoxide.

5,11,17,23-Tetra-*tert*-butyl-25,26,27,28-tetrakis[(*N*-(3,3-dimethyl-3-{(ethoxycarbonyl[*S*-(1*H*-indol-3-yl)methyl]methyl)amidocarbonylmethylammonio}propyl)carbamoylmethoxy]-2,8,14,20-tetrathiacalix[4]arene tetrabromide (*cone-***8**). Yield: 0.397 g (95%), mp 155 °С. ^1^H NMR (400 MHz, 298 K, DMSO-*d*_6_) δ 1.06 (s, 36H, (CH_3_)_3_C), 1.09 (t, ^3^*J*_HH_ = 7.1 Hz, 12H, C*H*_3_CH_2_O–), 1.91 (m, 8H, NHCH_2_C*H*_2_CH_2_N^+^), 3.09 (s, 24Н, (CH_3_)_2_N^+^), 3.17 (m, 8H, CHC*H*_2Trp_), 3.22 (m, 8H, NHCH_2_CH_2_C*H*_2_N^+^), 3.47 (m, 8H, NHC*H*_2_CH_2_CH_2_N^+^), 4.03 (q, ^3^*J*_HH_ = 7.1 Hz, 8H, CH_3_C*H*_2_O–), 4.10 (m, 8H, N^+^C*H*_2_CO), 4.58 (m, 4H, NНC*H*СО), 4.82 (s, 8H, OCH_2_CO), 6.97–7.49 (m, 20H, ArH_Trp_), 7.40 (s, 8Н, ArH), 8.51, (br.t, 4Н, N*H*CH_2_CH_2_CH_2_N^+^), 9.06 (br.s, 4Н, СОN*H*CH), 10.92 (s, 4Н, NH_Trp_); ^13^C NMR (100 MHz, 298 K, DMSO-*d*_6_) δ 171.47, 168.79, 163.62, 158.41, 147.15, 136.56, 134.89, 128.60, 127.44, 124.48, 121.53, 118.96, 118.40, 111.97, 109.23, 74.71, 63.41, 62.25, 61.31, 53.88, 51.55, 35.86, 34.38, 31.19, 27.31, 23.10, 14.35; Anal calcd. for C_128_H_172_Br_4_N_16_O_20_S_4_: C, 56.88; H, 6.41; Br, 11.83; N, 8.29; S, 4.75; found: C, 56.80; H, 6.54; Br, 11.65; N, 8.19; S, 4.55; ESIMS: calcd for [M – 4 Br^−^]^4+^ *m*/*z* = 595.8, [M – 3 Br^−^]^3+^ *m*/*z* = 820.7, [M – 2 Br^−^]^2+^ *m*/*z* = 1271.5, [M – Br^−^]^+^ *m*/*z* = 2622.9, found *m*/*z* = 595.7, 820.6, 1270.5, 2622.3; IR_νmax_: 1095 (СОС), 1670 (С=О), 2964, 3199 (N–H).

5,11,17,23-Tetra-*tert*-butyl-25,26,27,28-tetrakis[(*N*-(3,3-dimethyl-3-{(ethoxycarbonyl[*S*-(1*H*-indol-3-yl)methyl]methyl)amidocarbonylmethylammonio}propyl)carbamoylmethoxy]-2,8,14,20-tetrathiacalix[4]arene tetrabromide (1,3*-alternate-***9**). Yield: 0.389 g (93%), mp 162 °С. ^1^H NMR (400 MHz, 298 K, DMSO-*d*_6_) δ 1.08 (t, ^3^*J*_HH_ = 7.1 Hz, 12H, C*H*_3_CH_2_O–), 1.19 (s, 36H, (CH_3_)_3_C), 1.91 (m, 8H, NHCH_2_C*H*_2_CH_2_N^+^), 3.10 (s, 24Н, (CH_3_)_2_N^+^), 3.13–3.23 (m, 16Н, CHC*H*_2Trp_, NHCH_2_CH_2_C*H*_2_N^+^), 3.47 (m, 8H, NHC*H*_2_CH_2_CH_2_N^+^), 4.01 (s, 8Н, ОСН_2_СО), 4.04 (q, ^3^*J*_HH_ = 7.1 Hz, 8H, CH_3_C*H*_2_O–), 4.10 (m, 8H, N^+^C*H*_2_CO), 4.60 (m, 4H, NHC*H*CO), 6.97–7.50 (m, 20H, ArH_Trp_), 7.59 (s, 8Н, ArH), 8.02 (br.t, 4Н, N*H*CH_2_CH_2_CH_2_N^+^), 9.08 (br.s, 4Н, СОN*H*CH), 10.92 (s, 4Н, NH_Trp_); ^13^C NMR (100 MHz, 298 K, DMSO-*d*_6_) δ 171.48, 167.90, 163.67, 157.61, 146.57, 136.58, 133.51, 128.08, 127.45, 124.52, 121.54, 118.99, 118.43, 111.99, 109.25, 71.44, 63.23, 62.28, 61.32, 53.91, 51.59, 36.29, 34.35, 31.29, 27.34, 23.10, 14.39; Anal calcd. for C_128_H_172_Br_4_N_16_O_20_S_4_: C, 56.88; H, 6.41; Br, 11.83; N, 8.29; S, 4.75; found: C, 56.62; H, 6.34; Br, 11.65; N, 8.19; S, 4.55; ESIMS: calcd for [M – 4 Br^−^]^4+^ *m*/*z* = 595.8, [M – 3 Br^−^]^3+^ *m*/*z* = 820.7, [M – 2 Br^−^]^2+^ *m*/*z* = 1271.5, found *m*/*z* = 595.7, 820.7, 1272.0. IR_νmax_: 1094 (СОС), 1675 (С=О), 3206 (N–H).

5,11,17,23-Tetra-*tert*-butyl-25,26,27,28-tetrakis[(*N*-(2,2-diethyl-2-{(ethoxycarbonyl[*S*-(1*H*-indol-3-yl)methyl]methyl)amidocarbonylmethylammonio}ethyl)carbamoylmethoxy]-2,8,14,20-tetrathiacalix[4]arene tetrabromide (*cone-***10**). Yield: 0.384 g (94%), mp 150 °С. ^1^H NMR (400 MHz, 298 K, DMSO-*d*_6_) δ 1.05 (s, 36H, (CH_3_)_3_C), 1.07 (br.t, 12H, C*H*_3_CH_2_O–), 1.21 (br.t, 24Н, (C*H*_3_CH_2_)_2_N^+^), 3.17 (m, 8Н, CHC*H*_2Trp_), 3.37–3.52 (m, 24Н, NHCH_2_C*H*_2_N^+^, N^+^C*H*_2_CH_3_), 3.60 (m, 8H, NHC*H*_2_CH_2_N^+^), 4.03 (q, ^3^*J*_HH_ = 7.1 Hz, 8H, CH_3_C*H*_2_O–), 4.11 (br.s, 8H, N^+^C*H*_2_CO), 4.59 (m, 4Н, NHC*H*CO), 4.85 (s, 8H, OCH_2_CO), 6.97–7.50 (m, 20H, ArH_Trp_), 7.38 (s, 8Н, ArH), 8.77 (br.t, 4Н, N*H*CH_2_CH_2_CH_2_N^+^), 9.15 (br.s, 4Н, СОN*H*CH), 10.92 (s, 4Н, NH_Trp_); ^13^C NMR (100 MHz, 298 K, DMSO-*d*_6_) δ 171.54, 169.51, 163.58, 157.87, 147.32, 136.61, 135.04, 128.59, 127.40, 124.58, 121.53, 118.98, 118.42, 111.99, 109.17, 74.29, 61.34, 56.92, 56.68, 55.78, 53.83, 34.42, 32.84, 31.16, 27.25, 14.36; Anal calcd. for C_132_H_180_Br_4_N_16_O_20_S_4_: C, 57.47; H, 6.58; Br, 11.59; N, 8.12; S, 4.65; found: C, 57.65; H, 6.24; Br, 11.35; N, 7.95; S, 4.78; ESIMS: calcd for [M – 4 Br^−^]^4+^ *m*/*z* = 609.8, [M – 3 Br^−^]^3+^ *m*/*z* = 839.7, [M – 2 Br^−^]^2+^ *m*/*z* = 1299.6, [M – Br^−^]^+^ *m*/*z* = 2679.0, found *m*/*z* = 610.0, 839.6, 1299.0, 2679.3; IR_νmax_: 1094 (СОС), 1675 (С=О), 3186 (N–H).

5,11,17,23-Tetra-*tert*-butyl-25,26,27,28-tetrakis[(*N*-(2,2-diethyl-2-{(ethoxycarbonyl[*S*-(1*H*-indol-3-yl)methyl]methyl)amidocarbonylmethylammonio}ethyl)carbamoylmethoxy]-2,8,14,20-tetrathiacalix[4]arene tetrabromide (1,3*-alternate-***11**). Yield: 0.372 g (91%), mp 157 °С. ^1^H NMR (400 MHz, 298 K, DMSO-*d*_6_) δ: 1.10 (br.t, 12H, C*H*_3_CH_2_O–), 1.19 (s, 36H, (CH_3_)_3_C), 1.21 (br.t, 24Н, (C*H*_3_CH_2_)_2_N^+^), 3.21 (s, 12Н, (CH_3_C*H*_2_)_2_N^+^), 3.16 (m, 8Н, CHC*H*_2Trp_), 3.37–3.47 (m, 24Н, NHCH_2_C*H*_2_N^+^, N^+^C*H*_2_CH_3_), 3.90 (s, 8H, OCH_2_CO), 3.99–4.14 (m, 24H, NHC*H*_2_CH_2_N^+^, CH_3_C*H*_2_O–, N^+^C*H*_2_CO), 4.62 (m, 4Н, NHC*H*CO), 6.98–7.50 (m, 20H, ArH_Trp_), 7.59 (s, 8Н, ArH), 8.22 (br.t, 4Н, N*H*CH_2_CH_2_CH_2_N^+^), 9.13 (br.s, 4Н, СОN*H*CH), 10.93 (s, 4Н, NH_Trp_); ^13^C NMR (100 MHz, 298 K, DMSO-*d*_6_) δ 171.44, 168.53, 163.54, 157.40, 146.50, 136.60, 133.51, 127.91, 127.40, 124.54, 121.52, 118.95, 118.38, 111.98, 109.20, 71.00, 61.34, 56.90, 56.42, 55.66, 53.79, 34.35, 32.50, 31.36, 27.26, 14.39; Anal. calcd. for C_132_H_180_Br_4_N_16_O_20_S_4_: C, 57.47; H, 6.58; Br, 11.59; N, 8.12; S, 4.65; found: C, 57.12; H, 6.24; Br, 11.75; N, 7.89; S, 4.55; ESIMS: calcd for [M – 3 Br^−^]^3+^ *m*/*z* = 839.7, [M – 2 Br^−^]^2+^ *m*/*z* = 1299.6, found 839.5, 1300.6; IR_νmax_: 1092 (СОС), 1675 (С=О), 3186 (N–H).

### Determination of the hydrodynamic diameter by DLS

The particle diameter of thiacalix[4]arenes **8**–**11** was determined using a Zetasizer Nano ZS instrument. Deionized water was obtained using a Millipore-Q purification system. During the experiments, the concentration of the compounds varied from 1 × 10^−4^ to 1 × 10^−6^ М. The determination of the particle diameter was carried out over 1 h after the sample preparation. To assess the kinetic stability of the systems, the measurements were also carried out under similar conditions after 3 h and 5 h.

### Characterization of fluorescent properties by fluorescence spectroscopy

The fluorescence spectra were recorded on a fluorescent spectrometer Fluorolog 3 (Horiba Jobin Yvon). The excitation wavelength was chosen to be 280 nm for compounds **8**–**11**. The excitation and emission slits were set at 5 nm. Quartz cells with an optical path length of 10 mm were used. A correction of the emission spectra was carried out automatically by the software "Fluorescence". The spectra were recorded after a 10-minute exposure.

### Determination of particle diameter by TEM

The analysis of samples was carried out in a transmission electron microscope (TEM) by Hitachi (HT7700 Exalens). The sample preparation was as follows: samples of compounds **8–11** (1 × 10^−4^ М) were prepared similarly to those studied by DLS. 10 µL of the suspension was placed on a carbon-coated 3 mm copper grid, and drying was performed at room temperature. After drying, the grid was placed in a transmission electron microscope. The analysis was performed at an accelerating voltage of 100 kV in TEM mode.

### Determination of particle diameter by SEM

Additional measurements of the particle diameter were carried out by using a field-emission high-resolution scanning electron microscope (SEM) by Merlin Carl Zeiss. Observations of the morphology of the surface were made by applying an accelerating voltage of incident electrons at 5 kV and a current probe at 300 pA in order to minimize modifications to sample. The sample preparation was as follows: samples of compounds **8**–**11** (1 × 10^−4^ М) were prepared similar to those studied by DLS. The sample on the chuck was moved in the vacuum chamber apparatus by Quorum (Q 150T ES). A conductive layer was deposited by the cathode sputtering technique using an Au/Pd alloy (80/20). The thickness of the alloy was 15 nm.

## Supporting Information

File 1 Additional experimental parameters and results.

## References

[1] Salata O V (2004). J Nanobiotechnol.

[2] Chinen A B, Guan C M, Ferrer J R, Barnaby S N, Merkel T J, Mirkin C A (2015). Chem Rev.

[3] Chatterjee D K, Gnanasammandhan M K, Zhang Y (2010). Small.

[4] Ruedas-Rama M J, Walters J D, Orte A, Hall E A H (2012). Anal Chim Acta.

[5] Boisselier E, Astruc D (2009). Chem Soc Rev.

[6] Jamieson T, Bakhshi R, Petrova D, Pocock R, Imani M, Seifalian A M (2007). Biomaterials.

[7] Pankhurst Q A, Thanh N K T, Jones S K, Dobson J (2009). J Phys D: Appl Phys.

[8] Pankhurst Q A, Connolly J, Jones S K, Dobson J (2003). J Phys D: Appl Phys.

[9] Singh A K, Kanchanapally R, Fan Z, Senapati D, Ray P C (2012). Chem Commun.

[10] Ma S-Y, Yeh Y-C (2015). Anal Methods.

[11] Zheng J, Zhang C, Dickson R M (2004). Phys Rev Lett.

[12] Xu Y, Suslick K S (2010). Adv Mater.

[13] Yamada M, Ootashiro Y, Kondo Y, Hamada F (2013). Tetrahedron Lett.

[14] Yamada M, Gandhi M R, Kunda U M R, Hamada F (2016). J Inclusion Phenom Macrocyclic Chem.

[15] Zheng D-D, Fu D-Y, Wu Y, Sun Y-L, Tan L-L, Zhou T, Ma S-Q, Zha X, Yang Y-W (2014). Chem Commun.

[16] Zhou Y, Li H, Yang Y-W (2015). Chin Chem Lett.

[17] Kunda U M R, Yamada M, Katagiri H, Hamada F (2016). RSC Adv.

[18] Masson E, Ling X, Joseph R, Kyeremeh-Mensaha L, Lu X (2012). RSC Adv.

[19] Challa R, Ahuja A, Ali J, Khar R K (2005). AAPS PharmSciTech.

[20] Yakimova L S, Shurpik D N, Gilmanova L H, Makhmutova A R, Rakhimbekova A, Stoikov I I (2016). Org Biomol Chem.

[21] Shurpik D N, Padnya P L, Makhmutova L I, Yakimova L S, Stoikov I I (2015). New J Chem.

[22] Yakimova L S, Puplampu J B, Vavilova A A, Stoikov I I, Taylor J C (2015). Chapter 4: Polyammonium Derivatives of (thia)Calix[4]Arene: Synthesis and Interaction with Nucleic Acids. Advances in Chemistry Research.

[23] Puplampu J B, Yakimova L S, Vavilova A A, Rizvanov I Kh, Stoikov I I (2015). Macroheterocycles.

[24] Stoikov I I, Yakimova L S, Puplampu J B, Vavilova A A (2016). Organic Nanoreactors From Molecular to Supramolecular Organic Compounds. Systems Based on Calixarenes as the Basis for the Creation of Catalysts and Nanocontainers.

[25] Shurpik D N, Yakimova L S, Rizvanov I Kh, Plemenkov V V, Stoikov I I (2015). Macroheterocycles.

[26] Andreyko E A, Padnya P L, Stoikov I I (2015). J Phys Org Chem.

[27] Andreyko E A, Padnya P L, Stoikov I I (2014). Colloids Surf, A.

[28] Padnya P L, Andreyko E A, Gorbatova P A, Parfenov V V, Rizvanov I Kh, Stoikov I I (2017). RSC Adv.

[29] Shurpik D N, Padnya P L, Basimova L T, Evtugin V G, Plemenkov V V, Stoikov I I (2015). Mendeleev Commun.

[30] Shurpik D N, Padnya P L, Evtugyn V G, Mukhametzyanov T A, Khannanov A A, Kutyreva M P, Stoikov I I (2016). RSC Adv.

[31] Nosov R V, Stoikov I I (2014). Macroheterocycles.

[32] Nazarova A A, Yakimova L S, Klochkov V V, Stoikov I I (2017). New J Chem.

[33] Nazarova A A, Shibaeva K S, Stoikov I I (2016). Phosphorus, Sulfur Silicon Relat Elem.

[34] Vavilova A A, Nosov R V, Yakimova L S, Antipin I S, Stoikov I I (2013). Macroheterocycles.

[35] Andreyko E A, Padnya P L, Daminova R R, Stoikov I I (2014). RSC Adv.

[36] Padnya P L, Andreyko E A, Mostovaya O A, Rizvanov I Kh, Stoikov I I (2015). Org Biomol Chem.

[37] Arimori S, Nagasaki T, Shinkai S (1995). J Chem Soc, Perkin Trans 2.

[38] Stoikov I I, Padnya P L, Andreyko E A, Puplampu J B (2017). Design and applications of supramolecular systems based on (thia)calixarene ammonium derivatives. Supramolecular systems: chemistry, types and applications.

[39] Kaverzneva E D, Zvorykina V K, Kiseleva V V (1970). Bull Acad Sci USSR, Div Chem Sci (Engl Transl).

[40] Dehsorkhi A, Castelletto V, Hamley I W (2014). J Pept Sci.

[41] Zakharova L Ya, Kashapov R R, Pashirova T N, Mirgorodskaya A B, Sinyashin O G (2016). Mendeleev Commun.

[42] Bhattacharjee S (2016). J Controlled Release.

[43] Lakowicz J R (2006). Principles of Fluorescence Spectroscopy.

[44] Galić N, Burić N, Tomaš R, Frkanec L, Tomišić V (2011). Supramol Chem.

[45] Bregović N, Cindro N, Frkanec L, Tomišić V (2016). Supramol Chem.

[46] Gaeta C, De Rosa M, Fruilo M, Soriente A, Neri P (2005). Tetrahedron: Asymmetry.

[47] Qing G-y, He Y-b, Wang F, Qin H-j, Hu C-g, Yang X (2007). Eur J Org Chem.

[48] Qing G-Y, Wang F, He Y-B, Hu C-G, Yang X (2008). Supramol Chem.

[49] Nenov A, Rivalta I, Mukamel S, Garavelli M (2014). Comput Theor Chem.

[50] Kamiichi K, Doi M, Nabae M, Ishida T, Inoue M (1987). J Chem Soc, Perkin Trans 2.

[51] Ishida T, Shibata M, Fujii K, Inoue M (1983). Biochemistry.

[52] Vavilova A A, Nosov R V, Mostovaya O A, Stoikov I I (2016). Macroheterocycles.

[53] Vavilova A A, Nosov R V, Yagarmina A N, Mostovaya O A, Antipin I S, Konovalov A I, Stoikov I I (2012). Macroheterocycles.

[54] Reichardt C, Welton T (2011). Solvents and Solvent Effects in Organic Chemistry.

[55] Nibu Y, Abe H, Mikami N, Ito M (1983). J Phys Chem.

[56] Omidyan R, Omidyan M, Mohamadzadeh A (2016). RSC Adv.

[57] Ten G N, Yakovleva A A, Berezin M K, Baranov V I (2013). Opt Spectrosc.

[58] Kelly S M, Jess T J, Price N C (2005). Biochim Biophys Acta.

