# Supplementary material for: Self-assembly of chiral fluorescent nanoparticles based on water-soluble L-tryptophan derivatives of p-tert-butylthiacalix[4]arene
Source: Beilstein J Nanotechnol. 2017 Sep 4;8:1825–35. doi: 10.3762/bjnano.8.184 (PMC5629409; doi:10.3762/bjnano.8.184)
Supplement: File 1 — Additional experimental parameters and results. [file Beilstein_J_Nanotechnol-08-1825-s001.pdf]

Supporting Information

for

Self-assembly of chiral fluorescent nanoparticles based on water-soluble L-tryptophan derivatives of *p*-*tert*-butylthiacalix[4]arene

Pavel L. Padnya[1,2], Irina A. Khripunova[1], Olga A. Mostovaya[1], Timur A. Mukhametzyanov[1], Vladimir G. Evtugyn[1], Vyacheslav V. Vorobev[1], Yuri N. Osin[1] and Ivan I. Stoikov[*1]

Address: [1]Kazan Federal University, 420008 Kremlevskaya, 18, Kazan, Russian Federation and [2]Peoples Friendship University of Russia (RUDN University), 117198 Miklukho-Maklaya St., 6, Moscow, Russian Federation

Email: Ivan I. Stoikov - Ivan.Stoikov@mail.ru

* Corresponding author

Additional experimental parameters and results

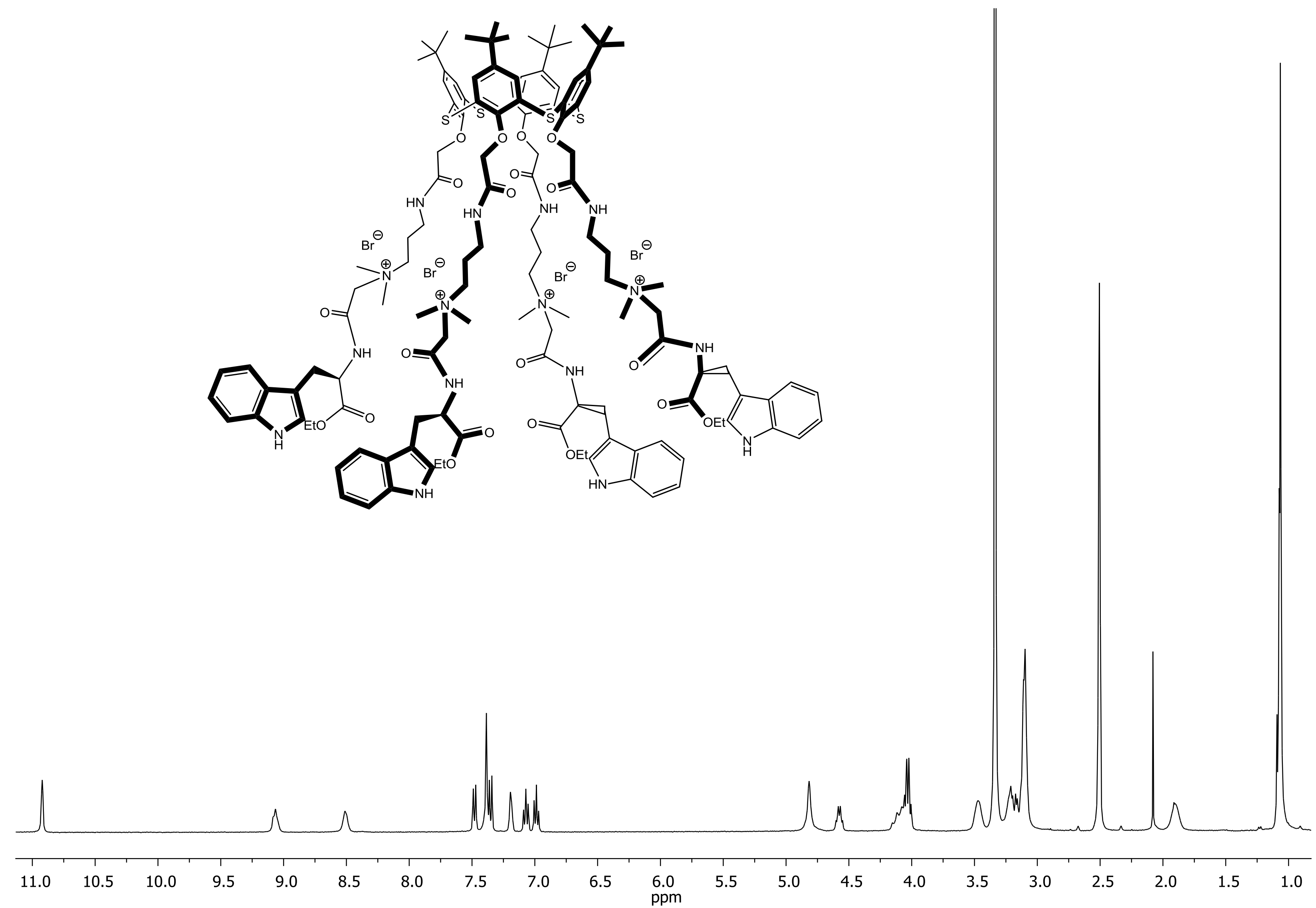

Figure S1: ^{1}H NMR spectrum of 5,11,17,23-tetra-*tert*-butyl-25,26,27,28-tetrakis[(*N*-(3’,3’-dimethyl-3’-{(ethoxycarbonyl[*S*-(1’’*H*-indol-3’’-yl)methyl]methyl)amidocarbonylmethyl}ammoniumpropyl)carbamoylmethoxy]-2,8,14,20-tetra thiacalix[4]arene tetrabromide (*cone*-**8**), DMSO-d_6, 298 K, 400 MHz

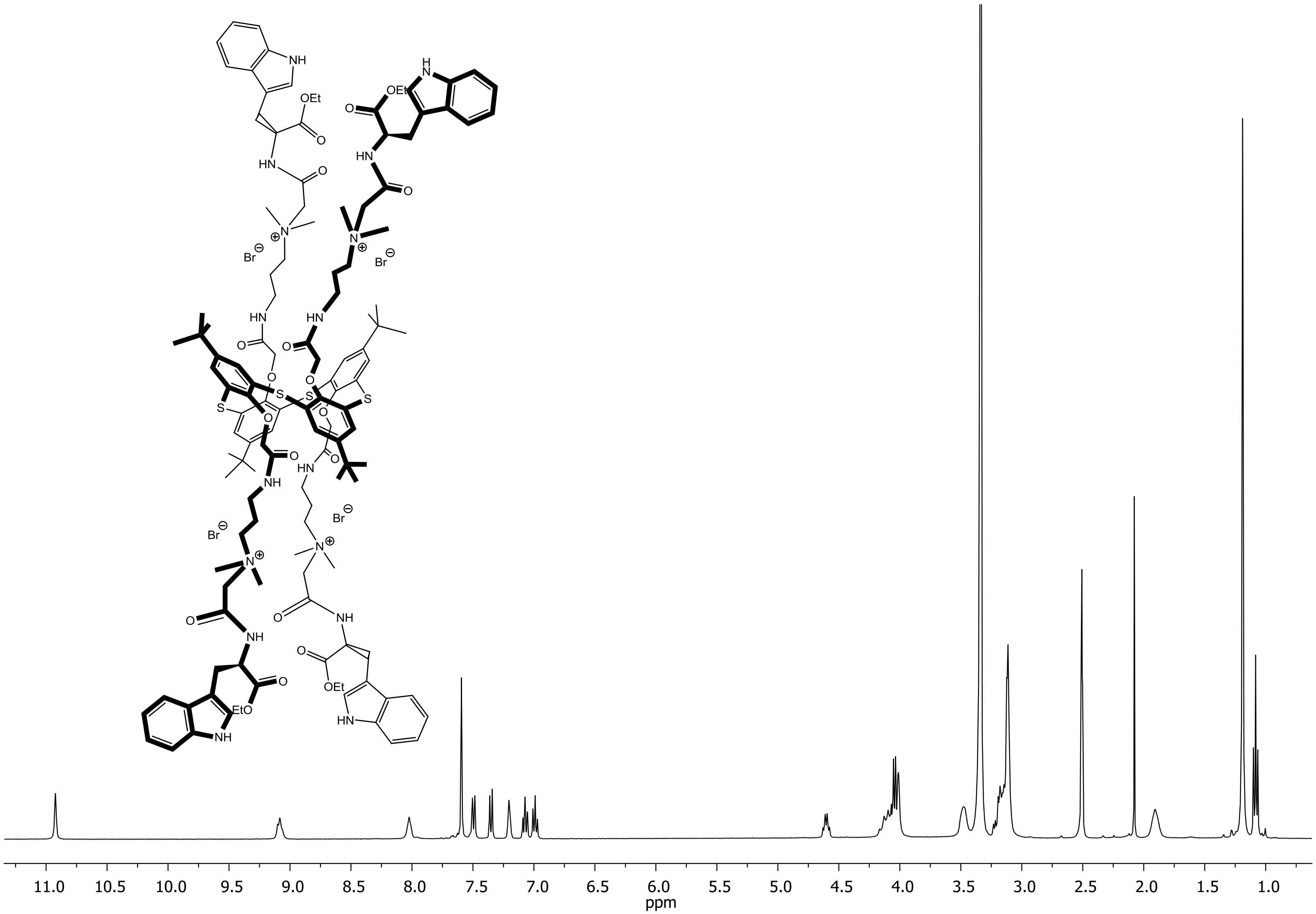

Figure S2: ^{1}H NMR spectrum of 5,11,17,23-tetra-*tert*-butyl-25,26,27,28-tetrakis[(*N*-(3',3'-dimethyl-3'-{(ethoxycarbonyl[*S*-(1''*H*-indol-3''-yl)methyl]methyl)amidocarbonylmethyl}ammoniumpropyl)carbamoylmethoxy]-2,8,14,20-tetra thiacalix[4]arene tetrabromide (1,3-*alternate*-**9**), DMSO-d_6, 298 K, 400 MHz.

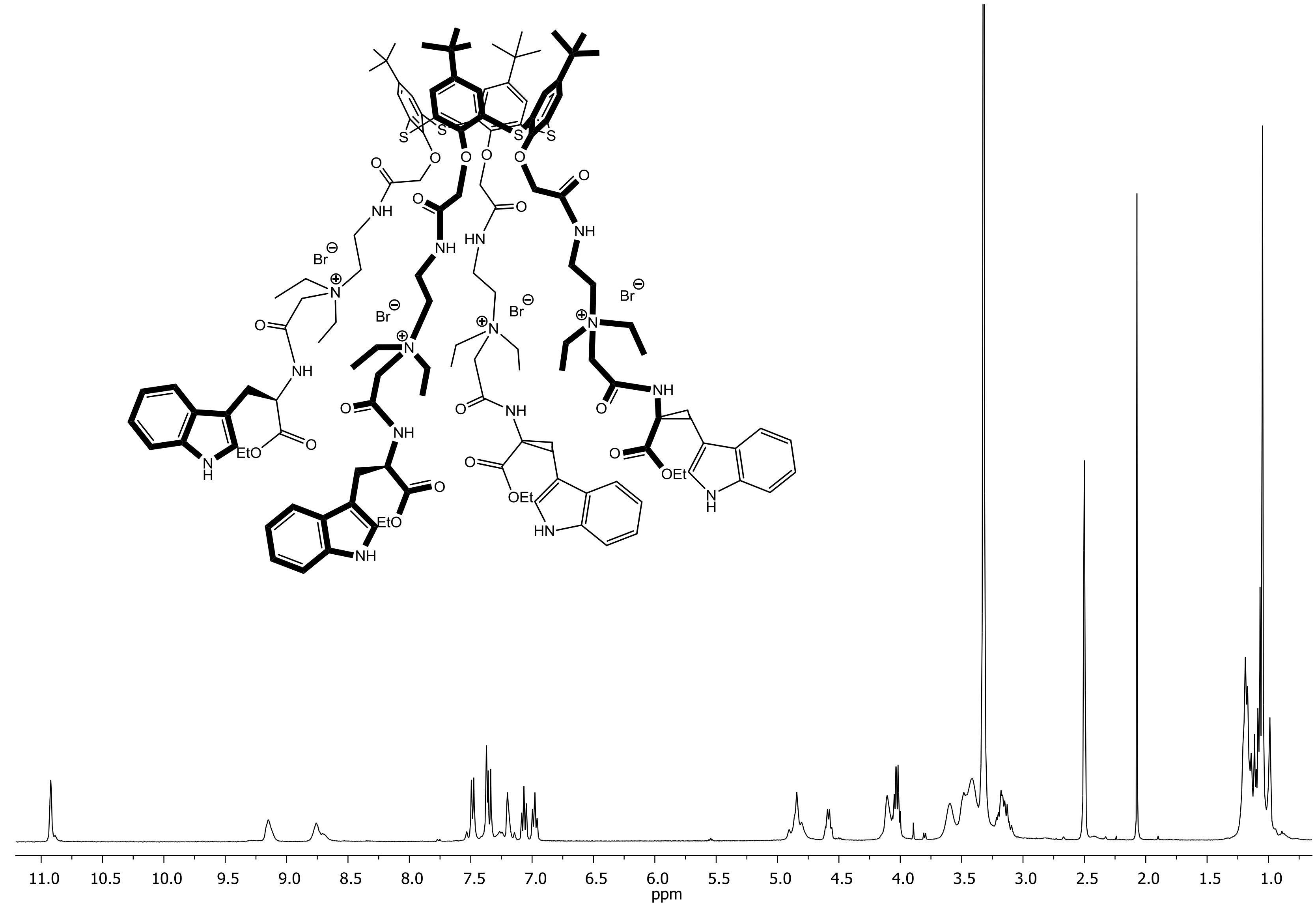

Figure S3: ^{1}H NMR spectrum of 5,11,17,23-tetra-*tert*-butyl-25,26,27,28-tetrakis[(*N*-(2',2'-diethyl-2'-{(ethoxycarbonyl[*S*-(1''*H*-indol-3''-yl)methyl]methyl)amidocarbonylmethyl}ammoniumethyl)carbamoylmethoxy]-2,8,14,20-tetra thiacalix[4]arene tetrabromide (*cone*-**10**), DMSO-d_6, 298 K, 400 MHz.

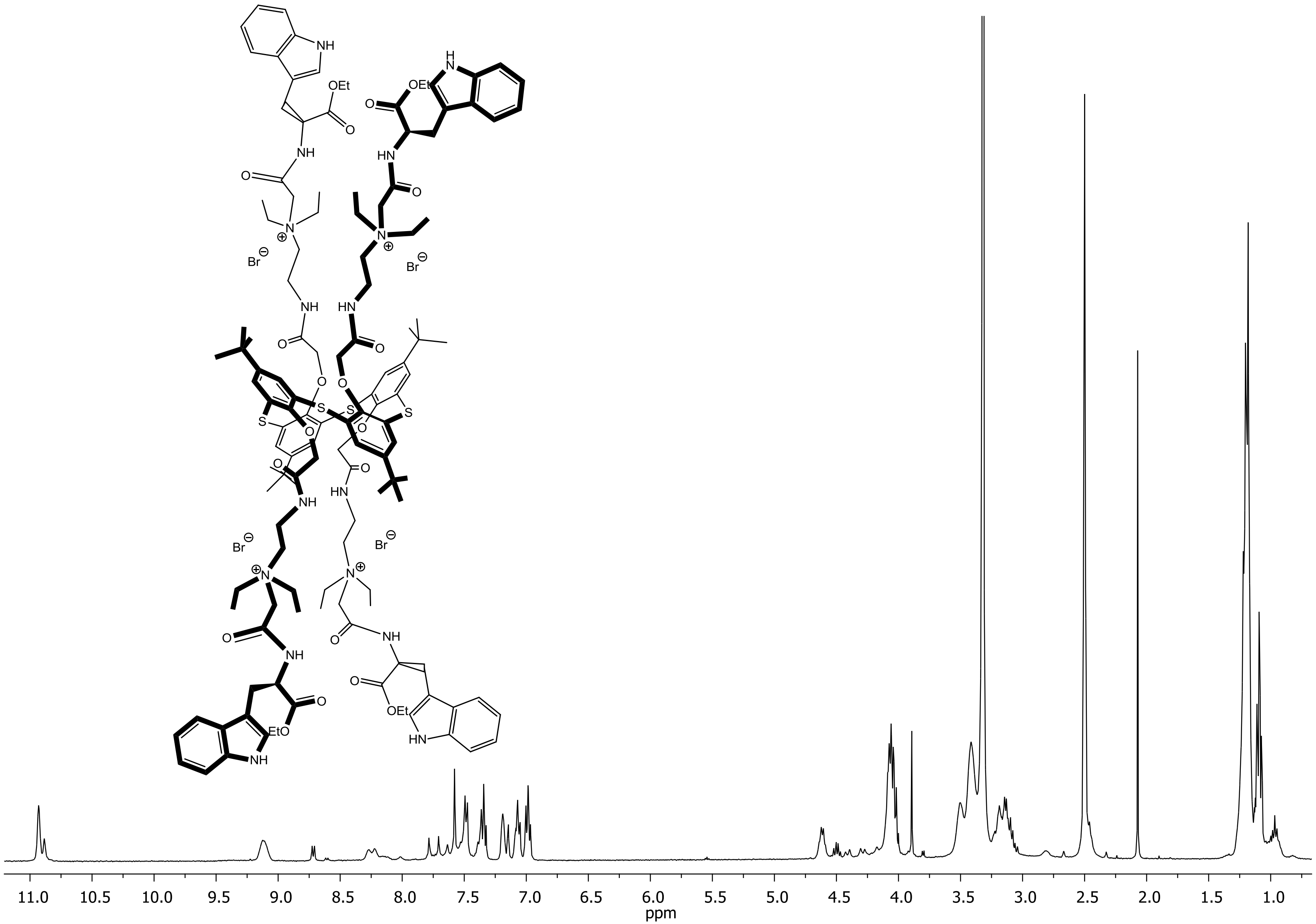

Figure S4: ^{1}H NMR spectrum of 5,11,17,23-tetra-*tert*-butyl-25,26,27,28-tetrakis[(*N*-(2',2'-diethyl-2'-{(ethoxycarbonyl[*S*-(1''*H*-indol-3''-yl)methyl]methyl)amidocarbonylmethyl}ammoniumethyl)carbamoylmethoxy]-2,8,14,20-tetra thiacalix[4]arene tetrabromide (1,3-*alternate*-**11**), DMSO-d_6, 298 K, 400 MHz.

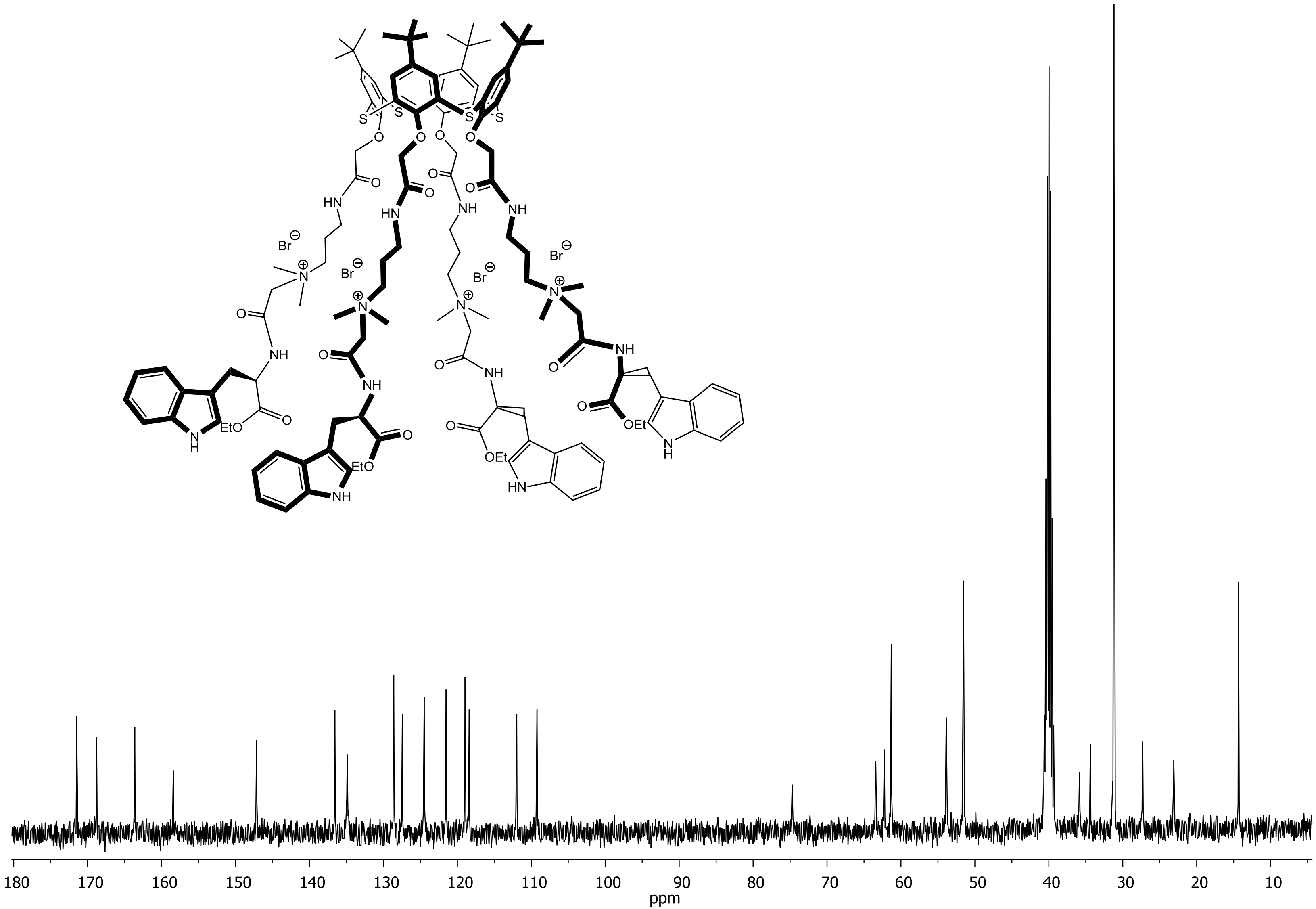

Figure S5: ^{13}C NMR spectrum of 5,11,17,23-tetra-*tert*-butyl-25,26,27,28-tetrakis[(*N*-(3’,3’-dimethyl-3’-{(ethoxycarbonyl[*S*-(1’’*H*-indol-3’’-yl)methyl]methyl)amidocarbonylmethyl}ammoniumpropyl)carbamoylmethoxy]-2,8,14,20-tetra thiacalix[4]arene tetrabromide (*cone*-**8**), DMSO-d_6, 298 K, 100 MHz.

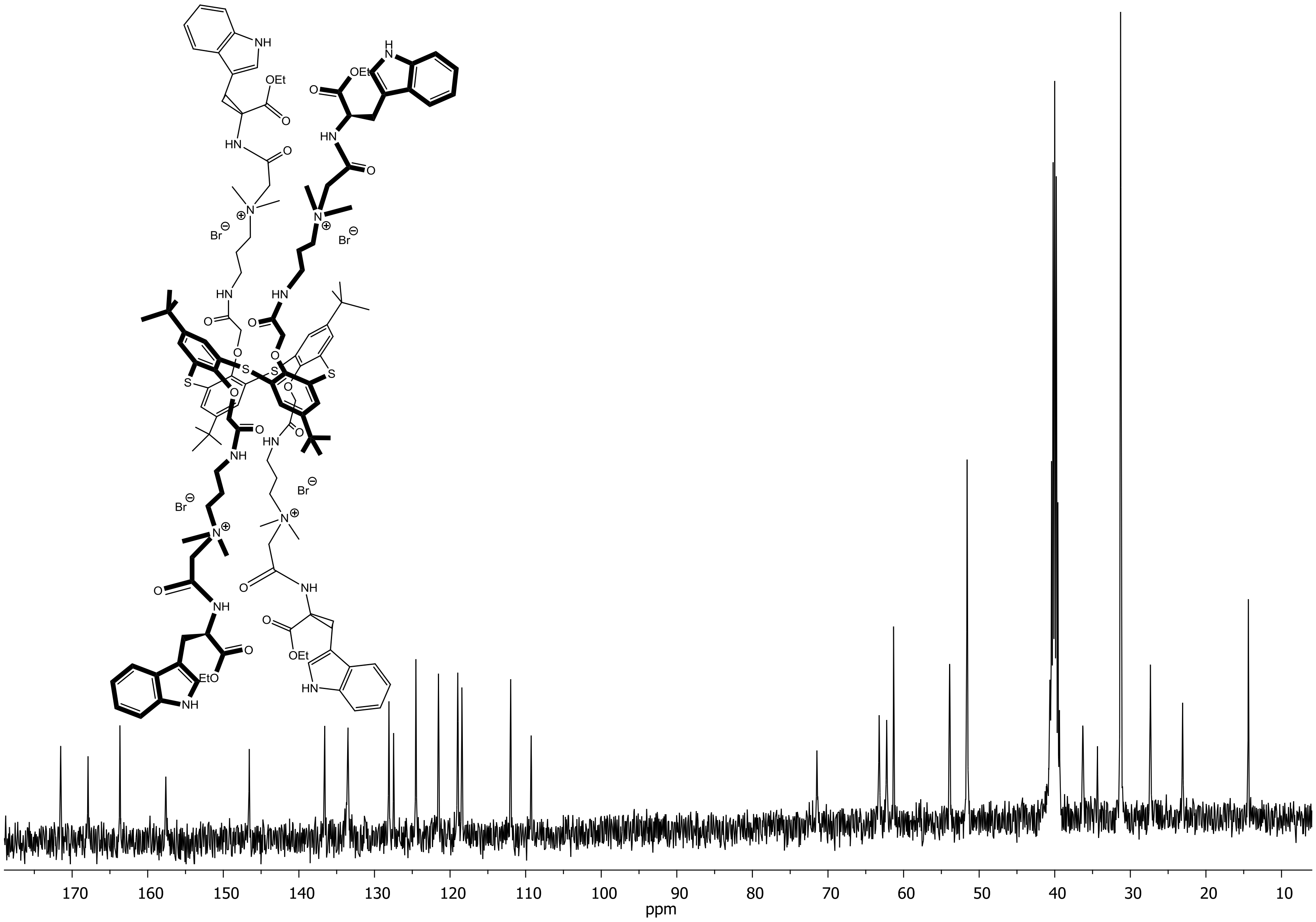

Figure S6: ^{13}C NMR spectrum of 5,11,17,23-tetra-*tert*-butyl-25,26,27,28-tetrakis[(*N*-(3’,3’-dimethyl-3’-{(ethoxycarbonyl[*S*-(1’’*H*-indol-3’’-yl)methyl]methyl)amidocarbonylmethyl}ammoniumpropyl)carbamoylmethoxy]-2,8,14,20-tetra thiacalix[4]arene tetrabromide (1,3-*alternate*-**9**), DMSO-d_6, 298 K, 100 MHz.

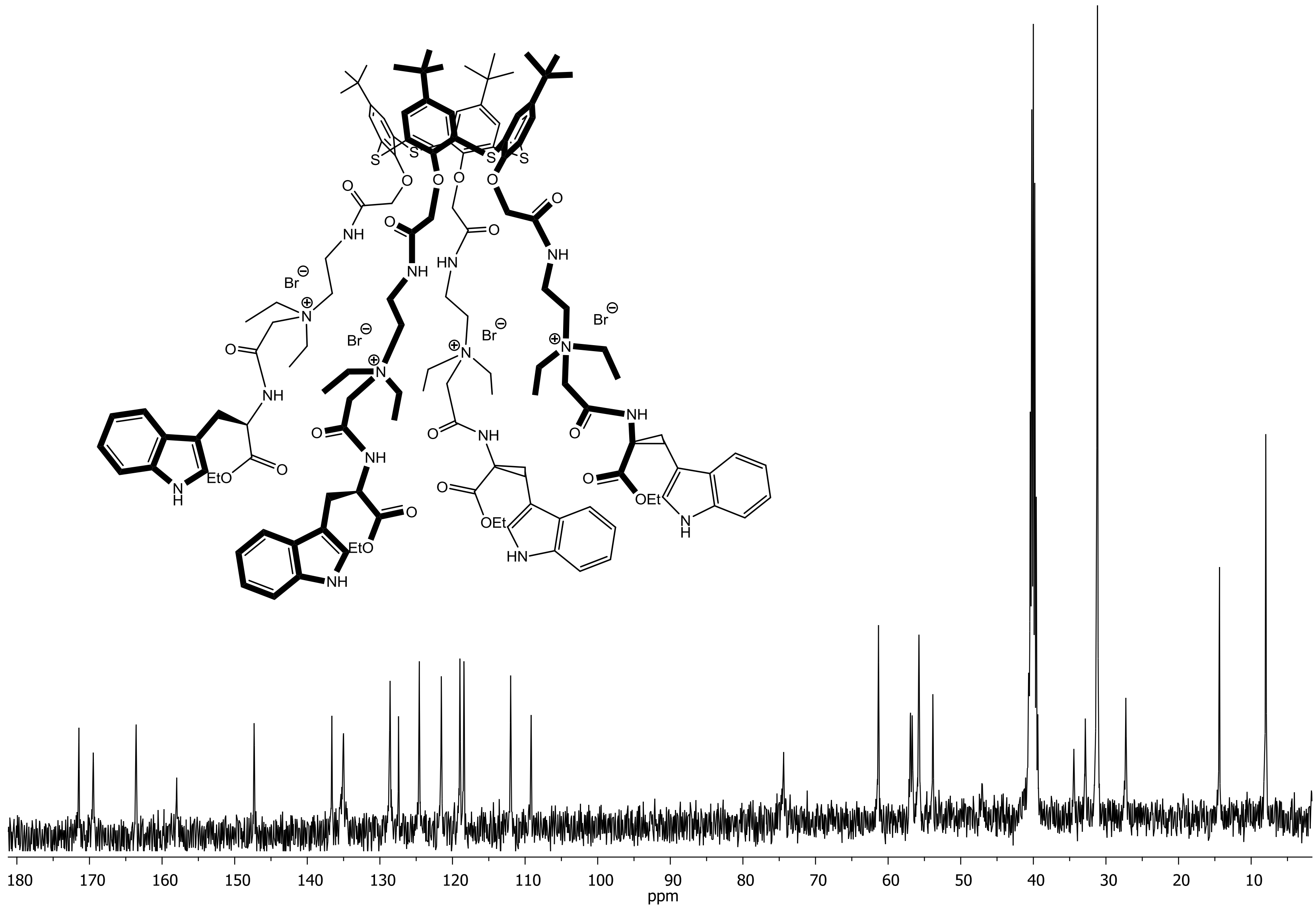

Figure S7: ^{13}C NMR spectrum of 5,11,17,23-tetra-*tert*-butyl-25,26,27,28-tetrakis[(*N*-(2’,2’-diethyl-2’-{(ethoxycarbonyl[*S*-(1’’*H*-indol-3’’-yl)methyl]methyl)amidocarbonylmethyl}ammoniumethyl)carbamoylmethoxy]-2,8,14,20-tetra thiacalix[4]arene tetrabromide (*cone*-**10**), DMSO-d_6, 298 K, 100 MHz.

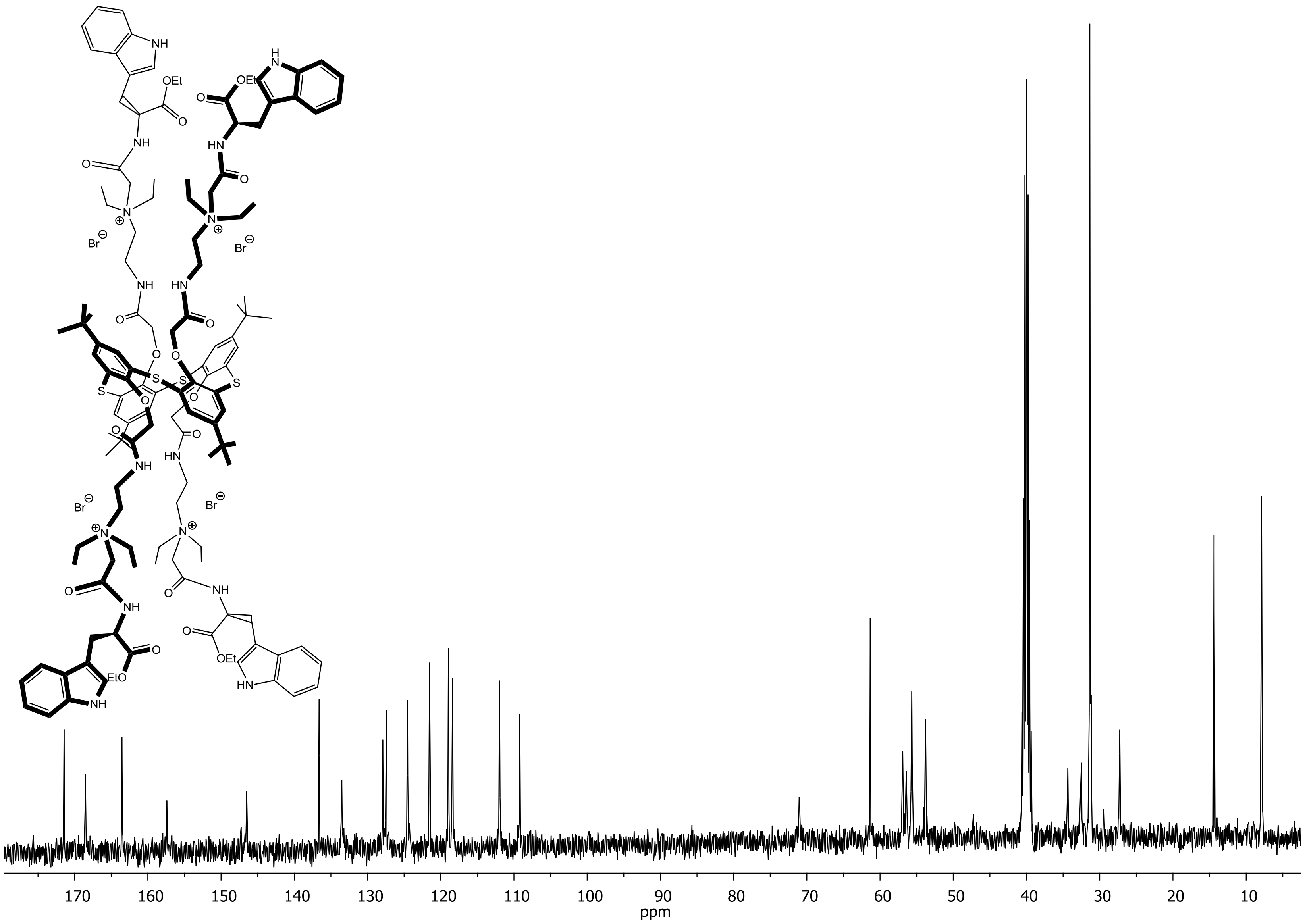

Figure S8: ^{13}C NMR spectrum of 5,11,17,23-tetra-*tert*-butyl-25,26,27,28-tetrakis[(*N*-(2',2'-diethyl-2'-{(ethoxycarbonyl[*S*-(1''*H*-indol-3''-yl)methyl]methyl)amidocarbonylmethyl}ammoniumethyl)carbamoylmethoxy]-2,8,14,20-tetra thiacalix[4]arene tetrabromide (1,3-*alternate*-**11**), DMSO-d_6, 298 K, 100 MHz.

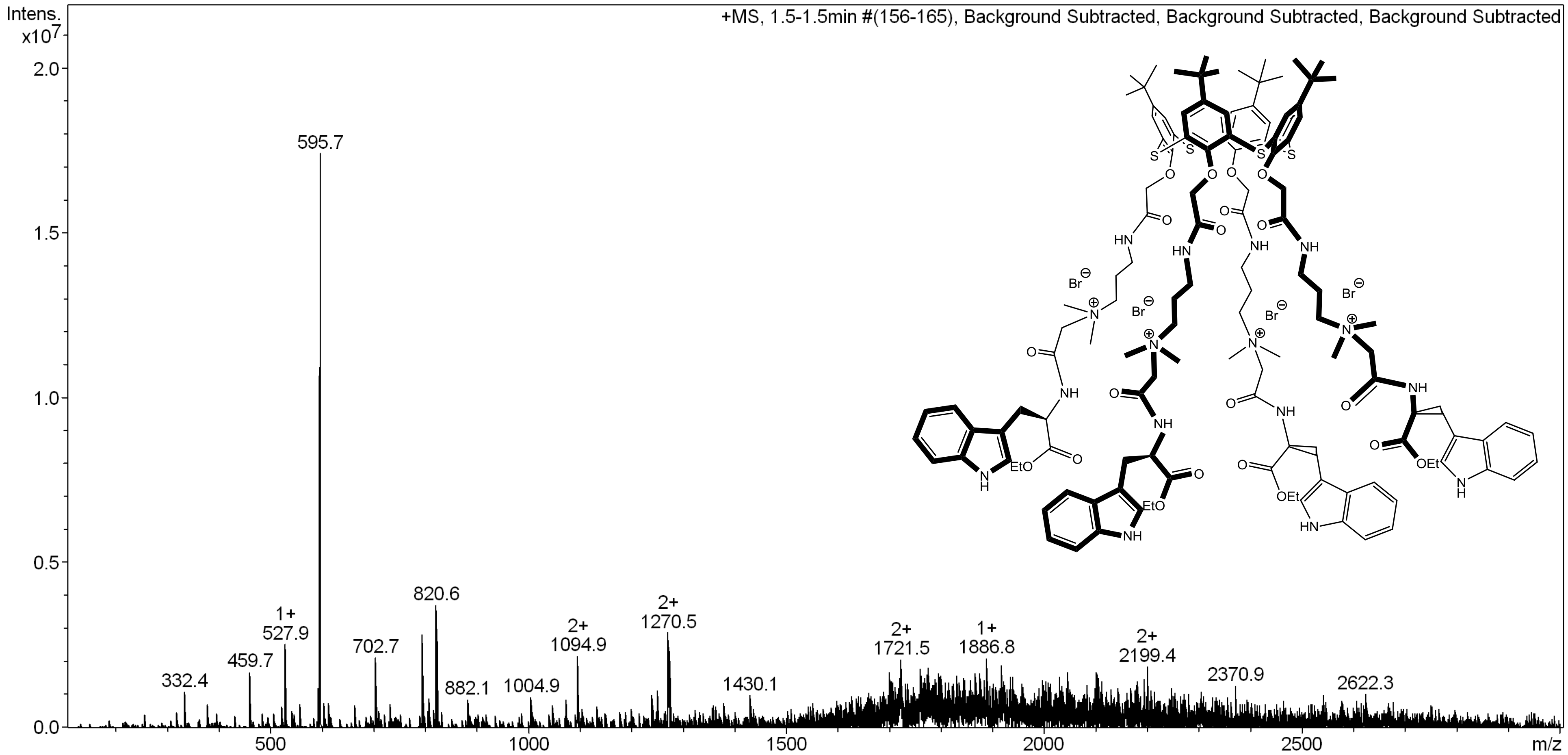

Figure S9: Mass spectrum (ESI) of 5,11,17,23-tetra-*tert*-butyl-25,26,27,28-tetrakis[(*N*-(3',3'-dimethyl-3'-{(ethoxycarbonyl[*S*-(1''*H*-indol-3''-yl)methyl]methyl)amidocarbonylmethyl}ammoniumpropyl)carbamoylmethoxy]-2,8,14,20-tetra thiacalix[4]arene tetrabromide (*cone*-**8**).

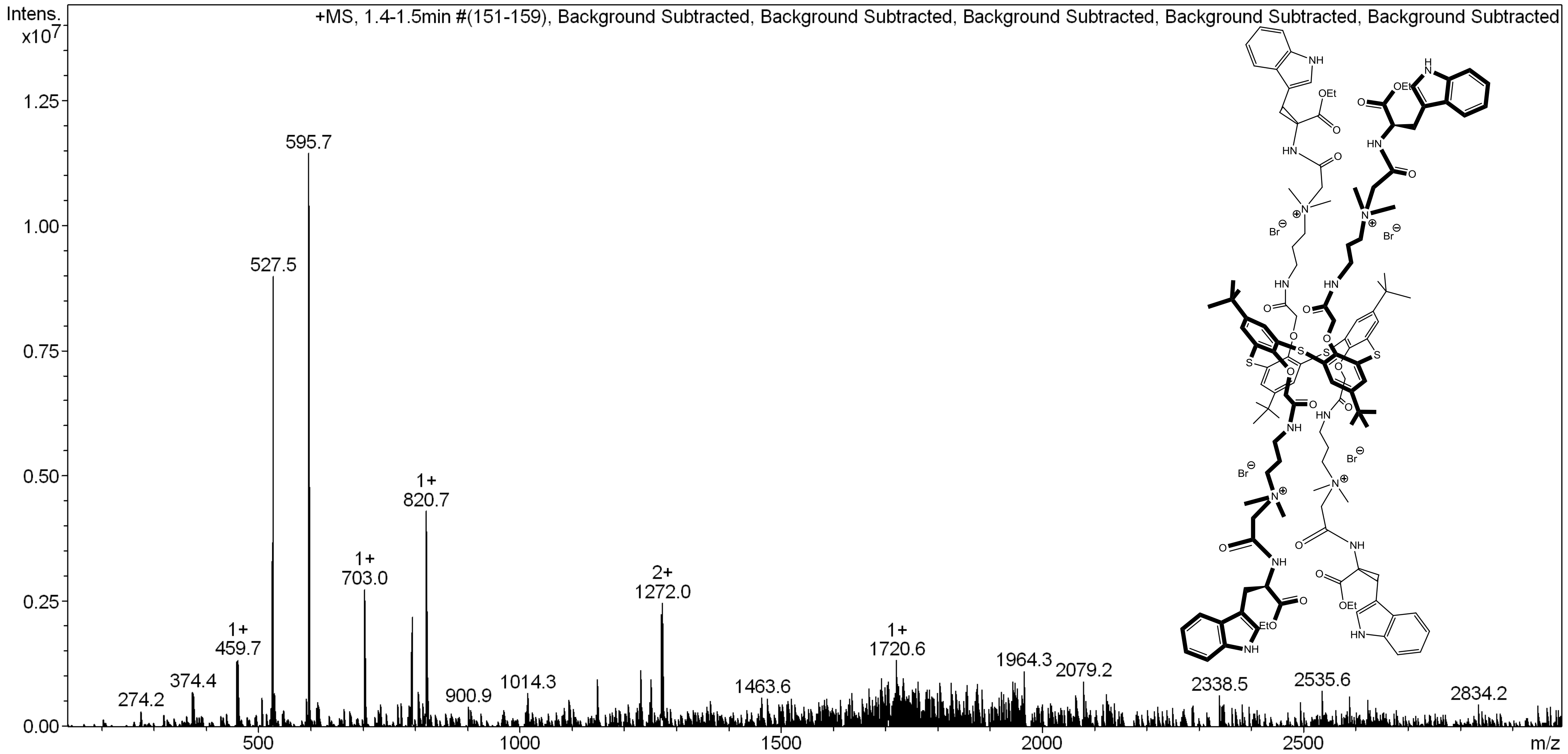

Figure S10: Mass spectrum (ESI) of 5,11,17,23-tetra-*tert*-butyl-25,26,27,28-tetrakis[(*N*-(3',3'-dimethyl-3'-{(ethoxycarbonyl[*S*-(1''*H*-indol-3''-yl)methyl]methyl)amidocarbonylmethyl}ammoniumpropyl)carbamoylmethoxy]-2,8,14,20-tetra thiacalix[4]arene tetrabromide (1,3-*alternate*-**9**).

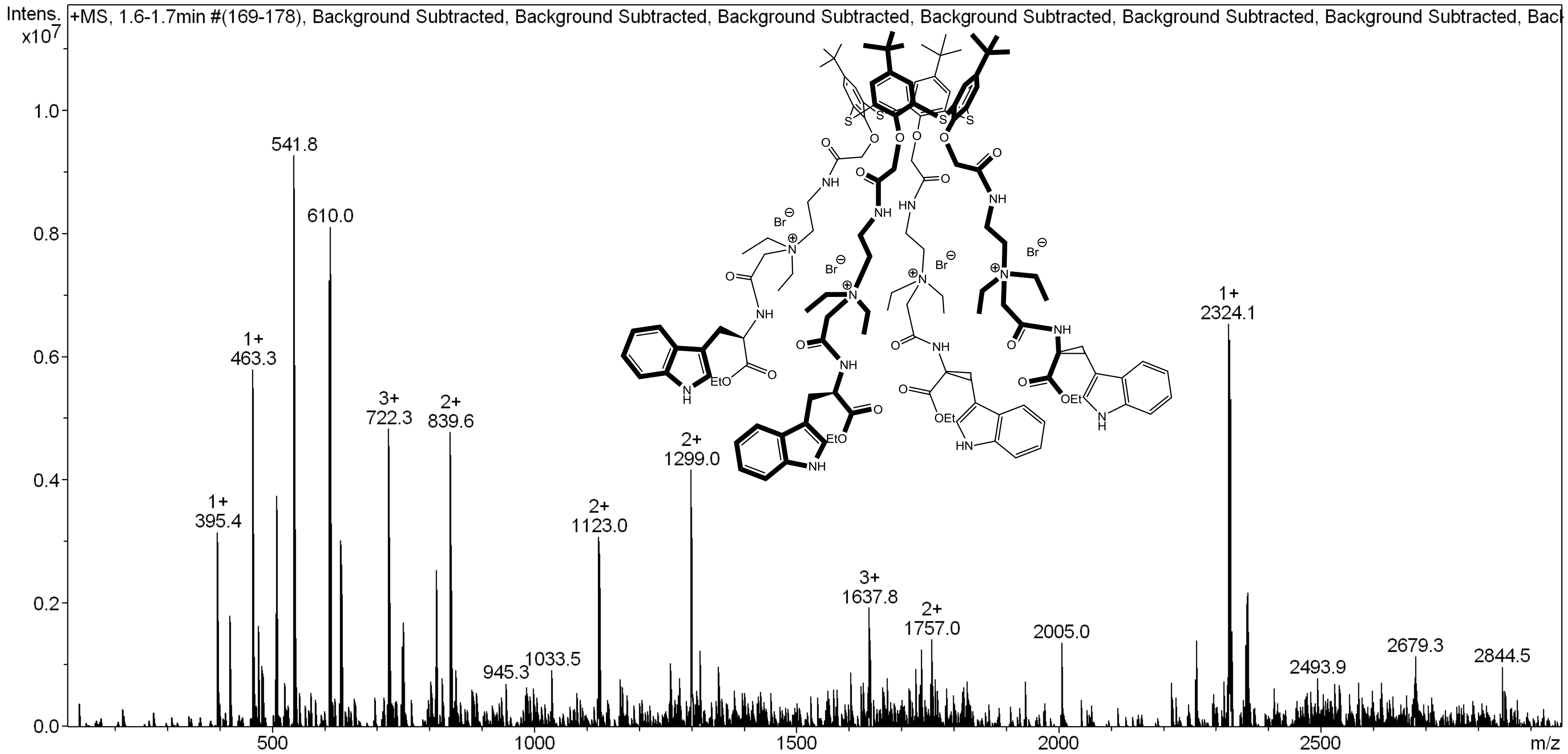

Figure S11: Mass spectrum (ESI) of 5,11,17,23-tetra-*tert*-butyl-25,26,27,28-tetrakis[(*N*-(2',2'-diethyl-2'-{(ethoxycarbonyl[*S*-(1''*H*-indol-3''-yl)methyl]methyl)amidocarbonylmethyl}ammoniumethyl)carbamoylmethoxy]-2,8,14,20-tetra thiacalix[4]arene tetrabromide (*cone*-**10**).

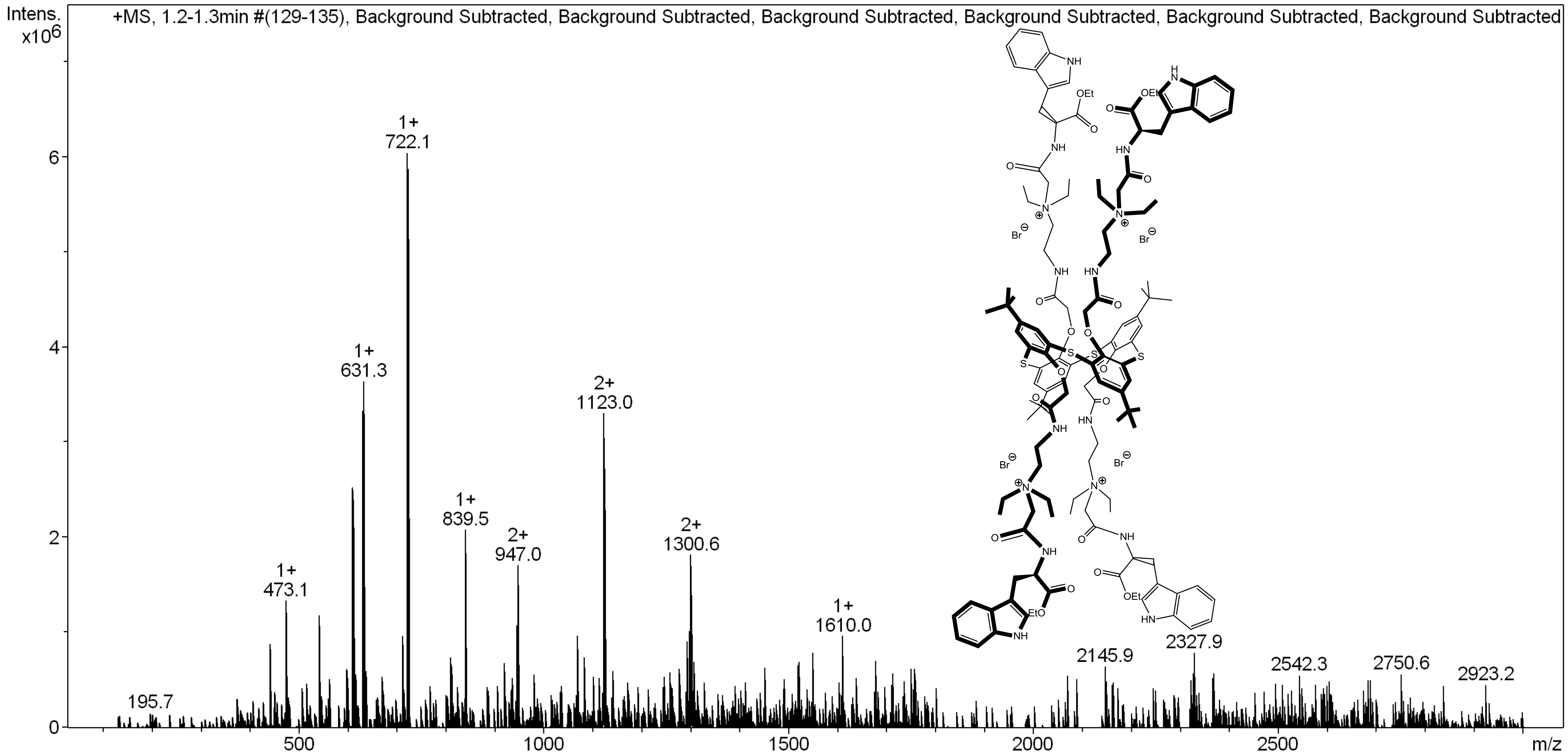

Figure S12: Mass spectrum (ESI) of 5,11,17,23-tetra-*tert*-butyl-25,26,27,28-tetrakis[(*N*-(2',2'-diethyl-2'-{(ethoxycarbonyl[*S*-(1''*H*-indol-3''-yl)methyl]methyl)amidocarbonylmethyl}ammoniumethyl)carbamoylmethoxy]-2,8,14,20-tetra thiacalix[4]arene tetrabromide (1,3-*alternate*-**11**).

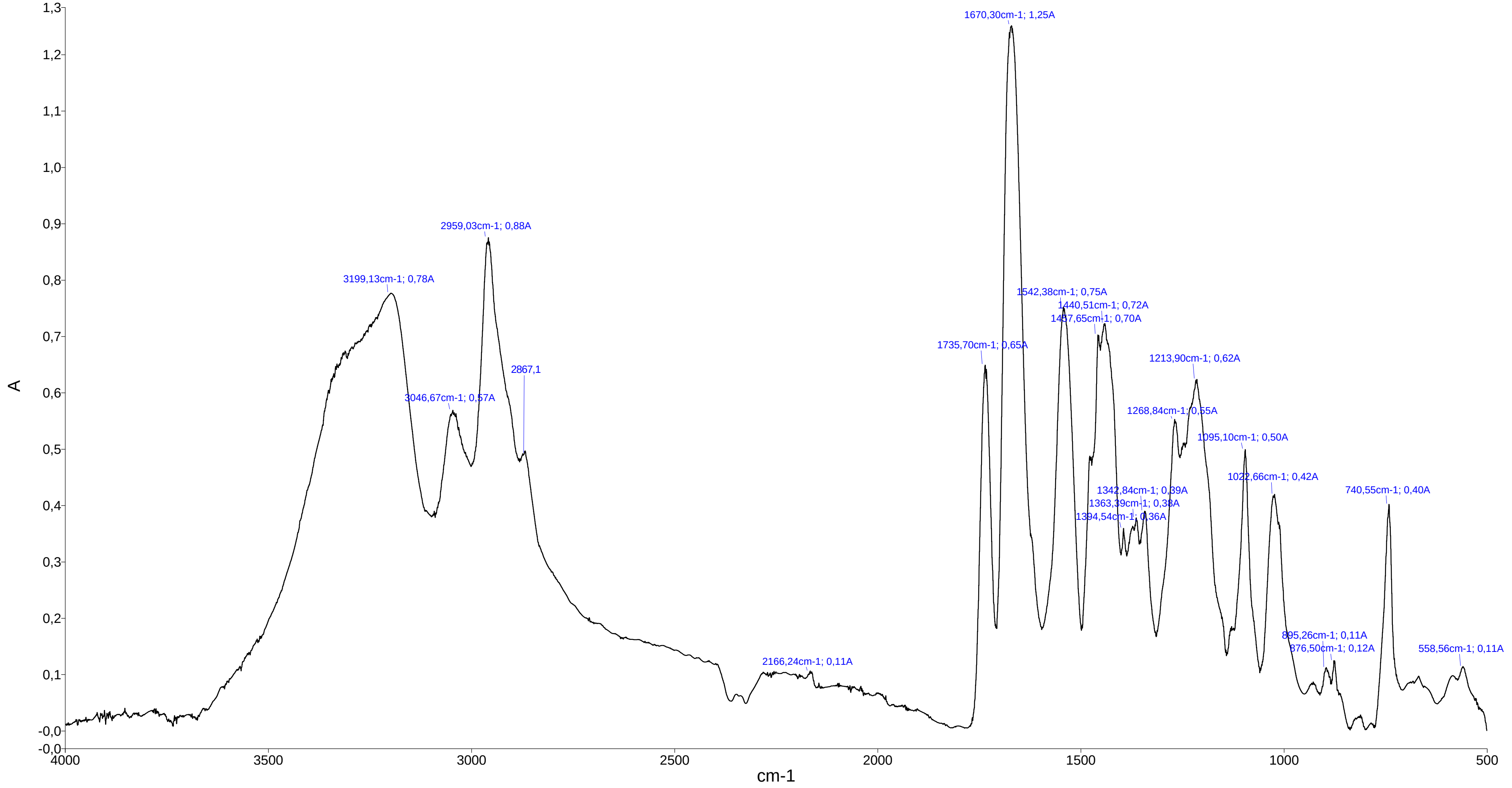

Figure S13: IR spectrum of 5,11,17,23-tetra-*tert*-butyl-25,26,27,28-tetrakis[(*N*-(3’,3’-dimethyl-3’-{(ethoxycarbonyl[*S*-(1’’*H*-indol-3’’-yl)methyl]methyl)amidocarbonylmethyl}ammoniumpropyl)carbamoylmethoxy]-2,8,14,20-tetra thiacalix[4]arene tetrabromide (*cone*-**8**).

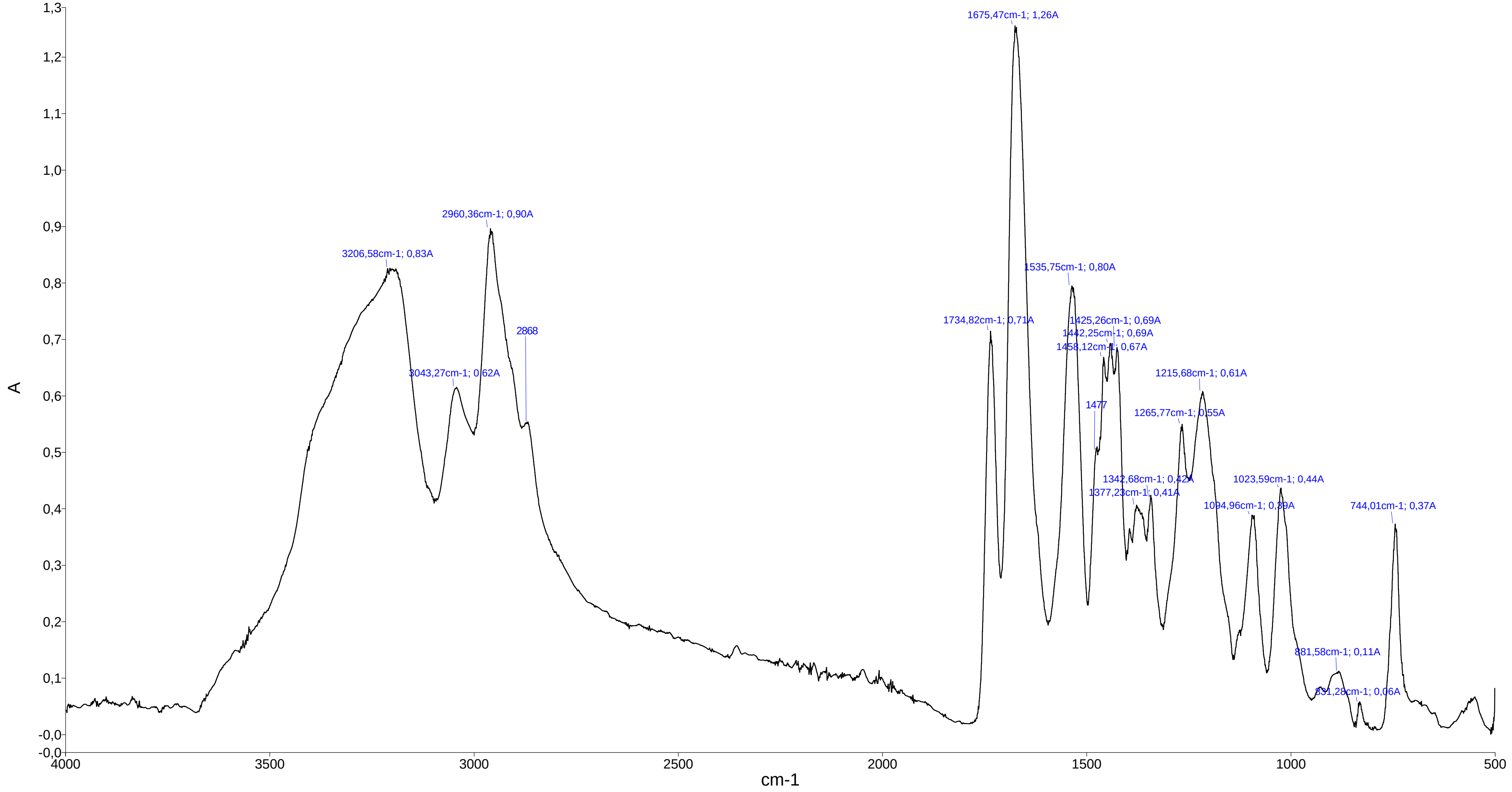

Figure S14: IR spectrum of 5,11,17,23-tetra-*tert*-butyl-25,26,27,28-tetrakis[(*N*-(3’,3’-dimethyl-3’-{(ethoxycarbonyl[*S*-(1’’*H*-indol-3’’-yl)methyl]methyl)amidocarbonylmethyl}ammoniumpropyl)carbamoylmethoxy]-2,8,14,20-tetra thiacalix[4]arene tetrabromide (1,3-*alternate*-**9**).

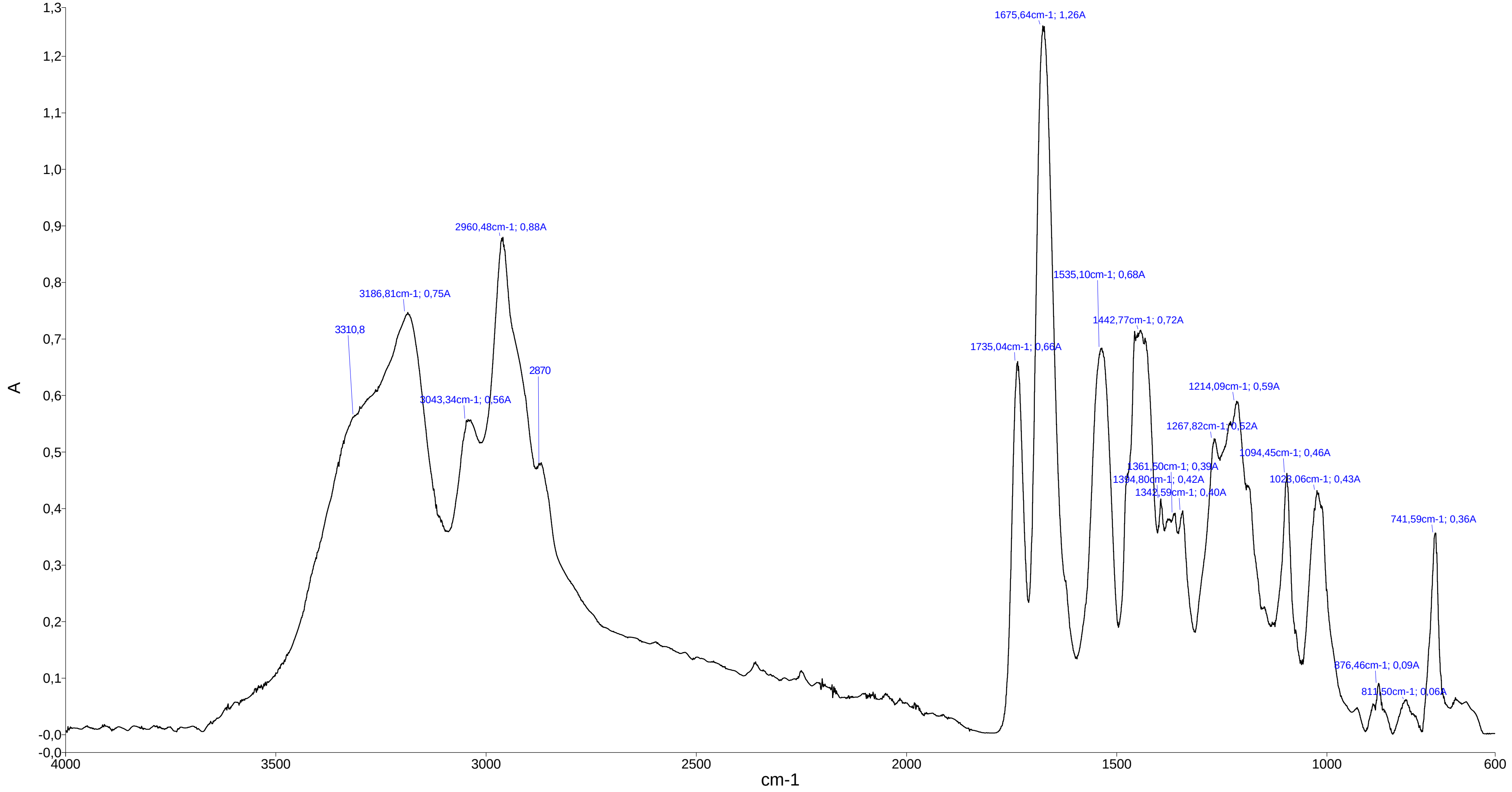

Figure S15: IR spectrum of 5,11,17,23-tetra-*tert*-butyl-25,26,27,28-tetrakis[(*N*-(2',2'-diethyl-2'-{(ethoxycarbonyl[*S*-(1''*H*-indol-3''-yl)methyl]methyl)amidocarbonylmethyl}ammoniumethyl)carbamoylmethoxy]-2,8,14,20-tetra thiacalix[4]arene tetrabromide (*cone*-**10**).

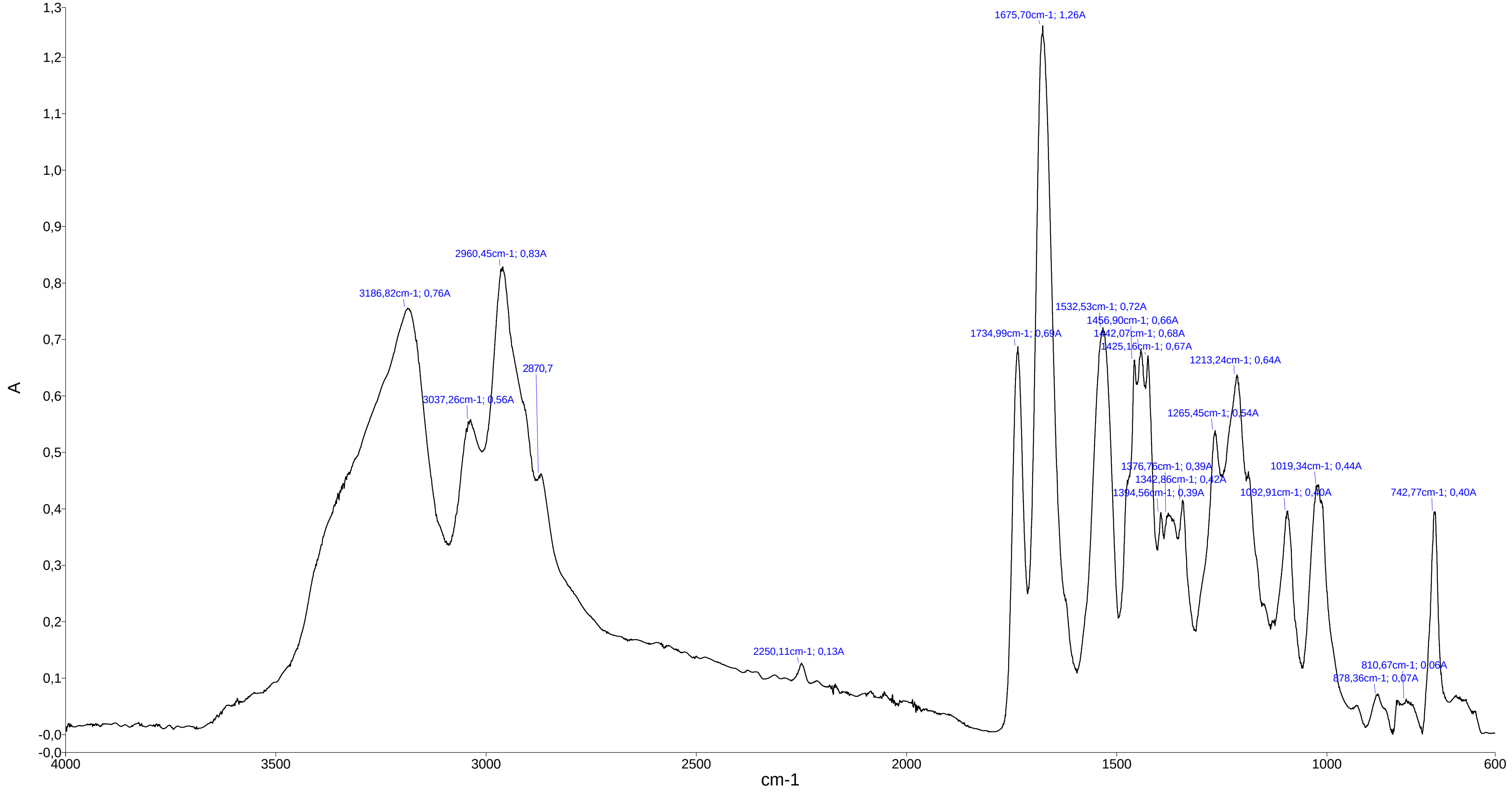

Figure S16: IR spectrum of 5,11,17,23-tetra-*tert*-butyl-25,26,27,28-tetrakis[(*N*-(2',2'-diethyl-2'-{(ethoxycarbonyl[*S*-(1''*H*-indol-3''-yl)methyl]methyl)amidocarbonylmethyl}ammoniumethyl)carbamoylmethoxy]-2,8,14,20-tetra thiacalix[4]arene tetrabromide (1,3-*alternate*-**11**).

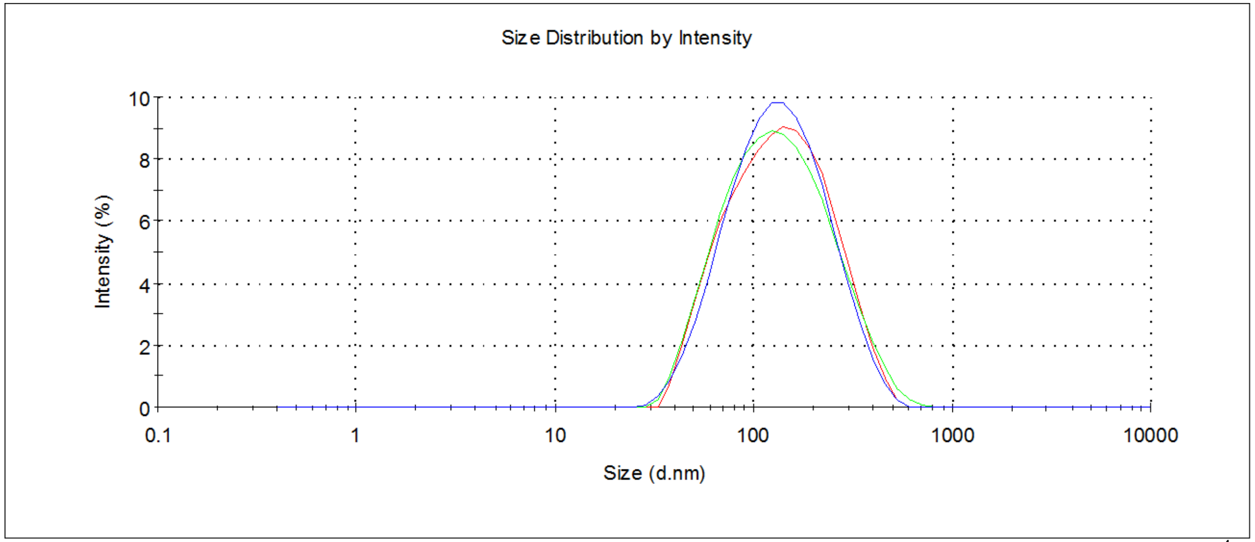

Figure S17: Size distribution by intensity of solution of the macrocycle **8** in the water (1×10^{-4} M).

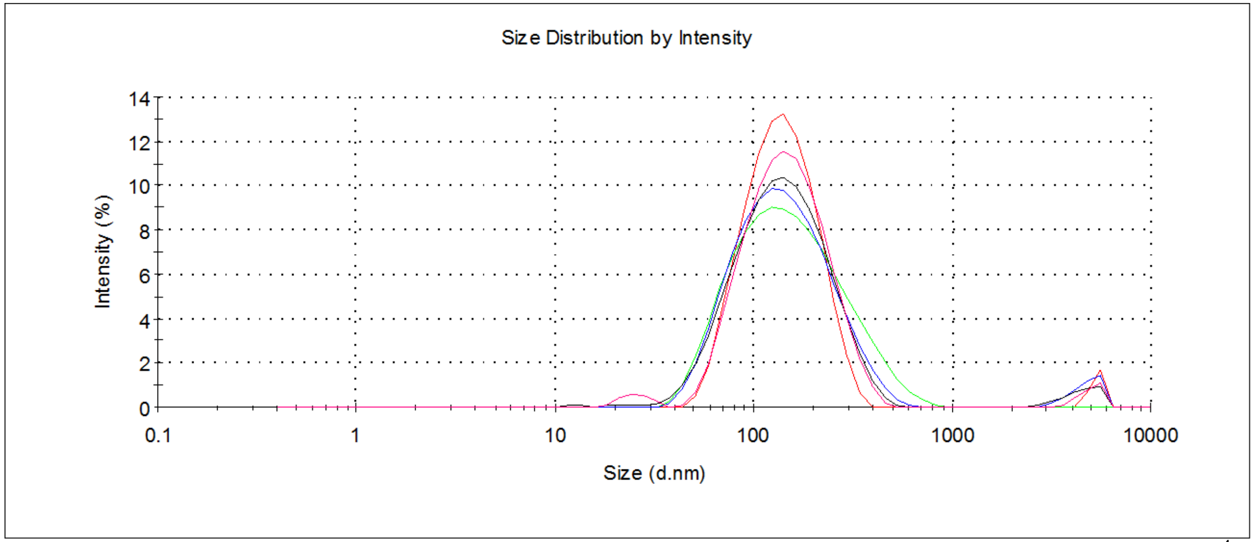

Figure S18: Size distribution by intensity of solution of the macrocycle **9** in the water (1 × 10^{-4} M).

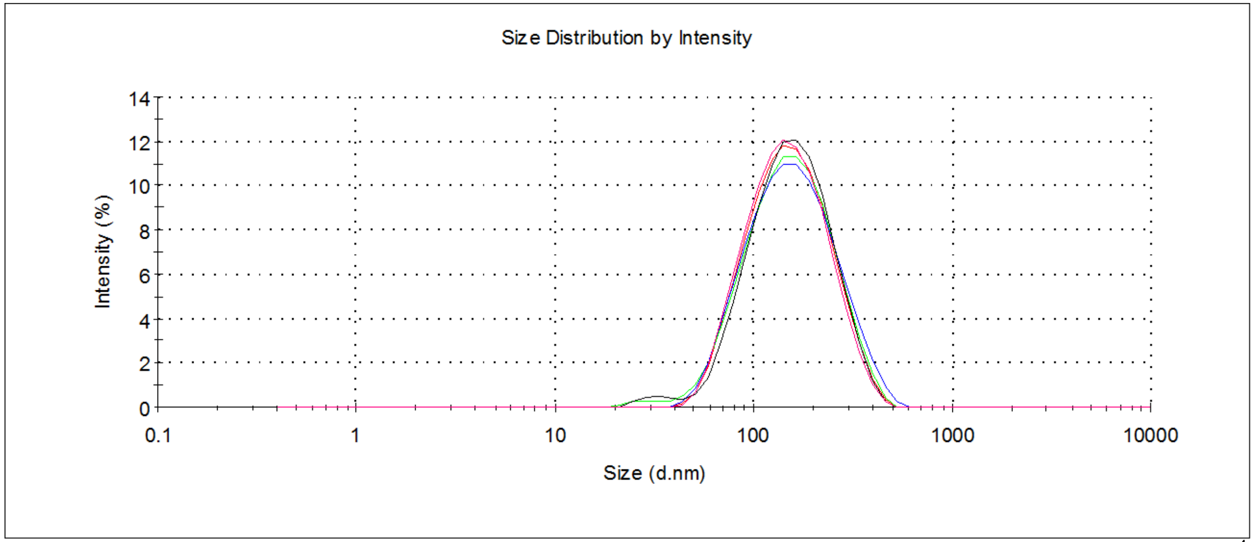

Figure S19: Size distribution by intensity of solution of the macrocycle **10** in the water (1×10^{-4} M).

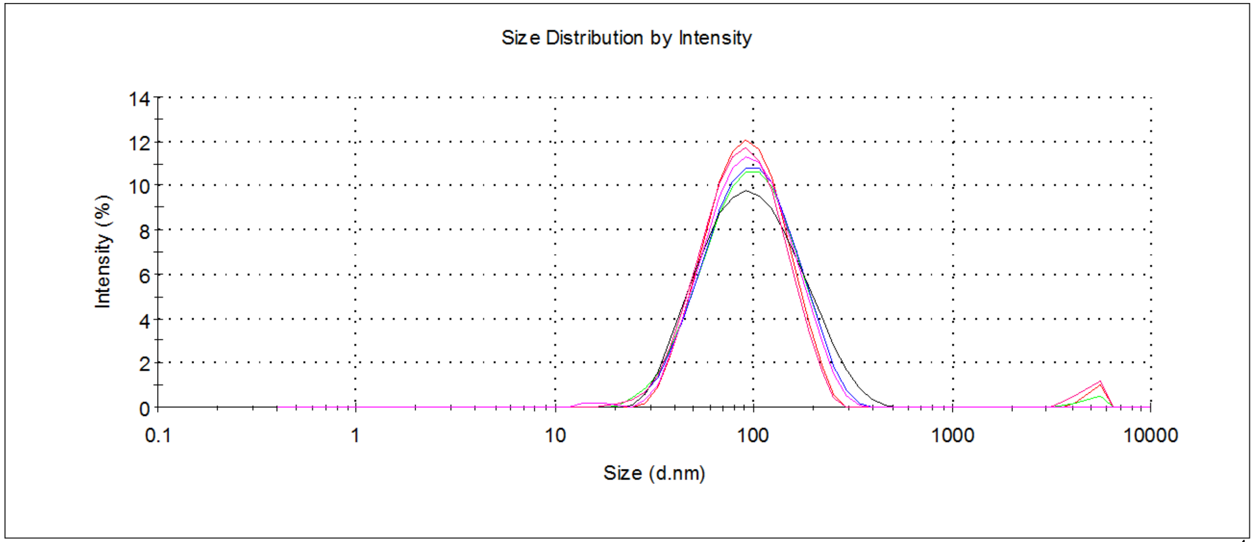

Figure S20: Size distribution by intensity of solution of the macrocycle **11** in the water (1×10^{-4} M).

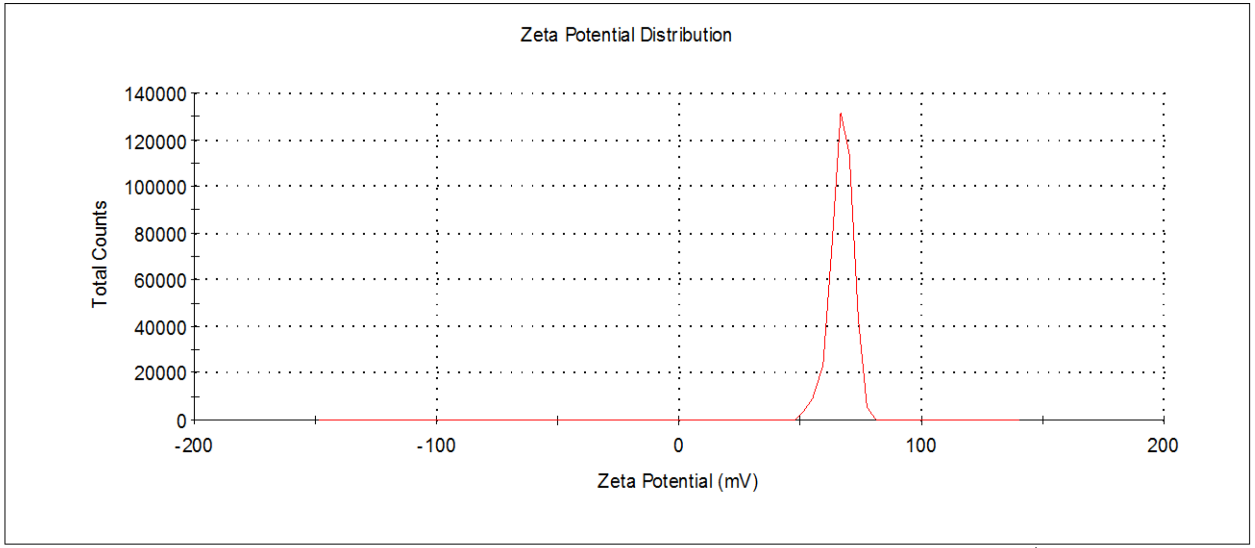

Figure S21: Zeta potential of solution of the macrocycle **8** in the water (1×10^{-4} M).

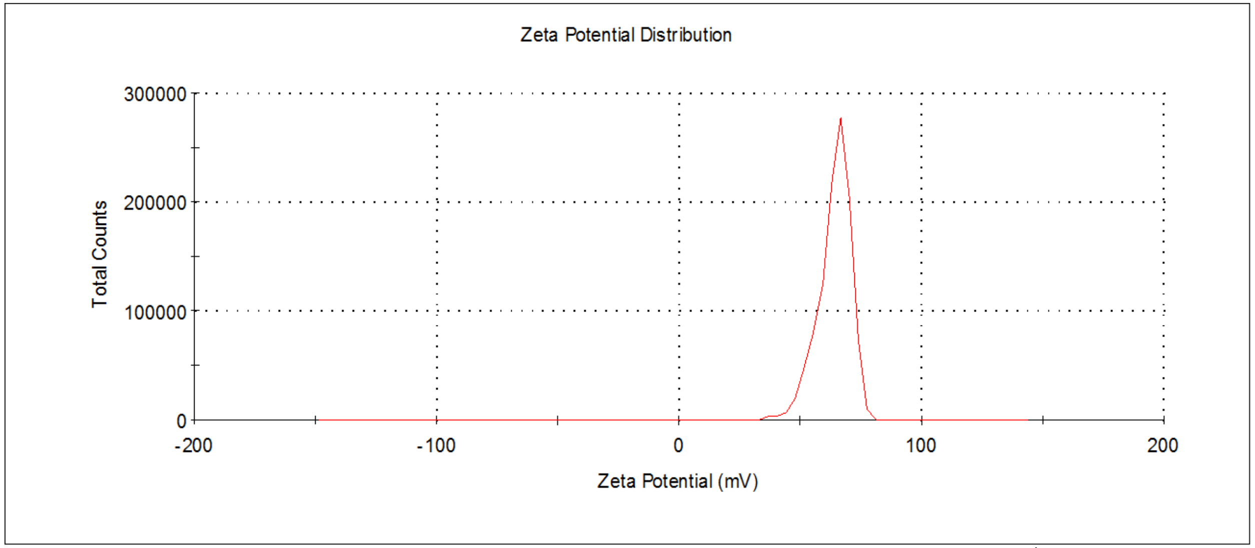

Figure S22: Zeta potential of solution of the macrocycle **9** in the water (1×10^{-4} M).

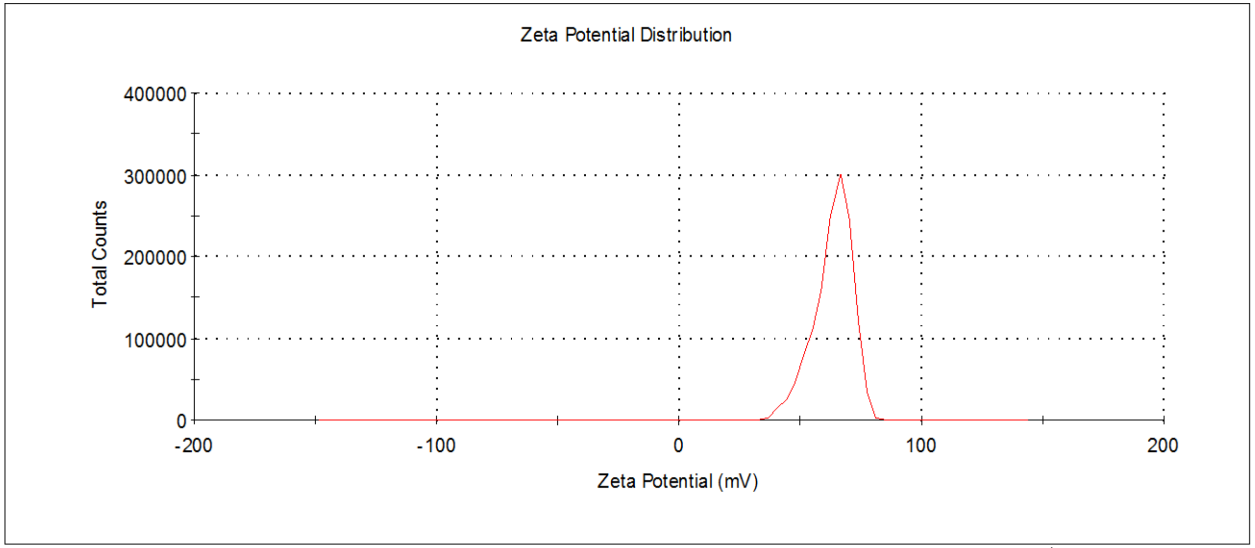

Figure S23: Zeta potential of solution of the macrocycle **10** in the water (1×10^{-4} M).

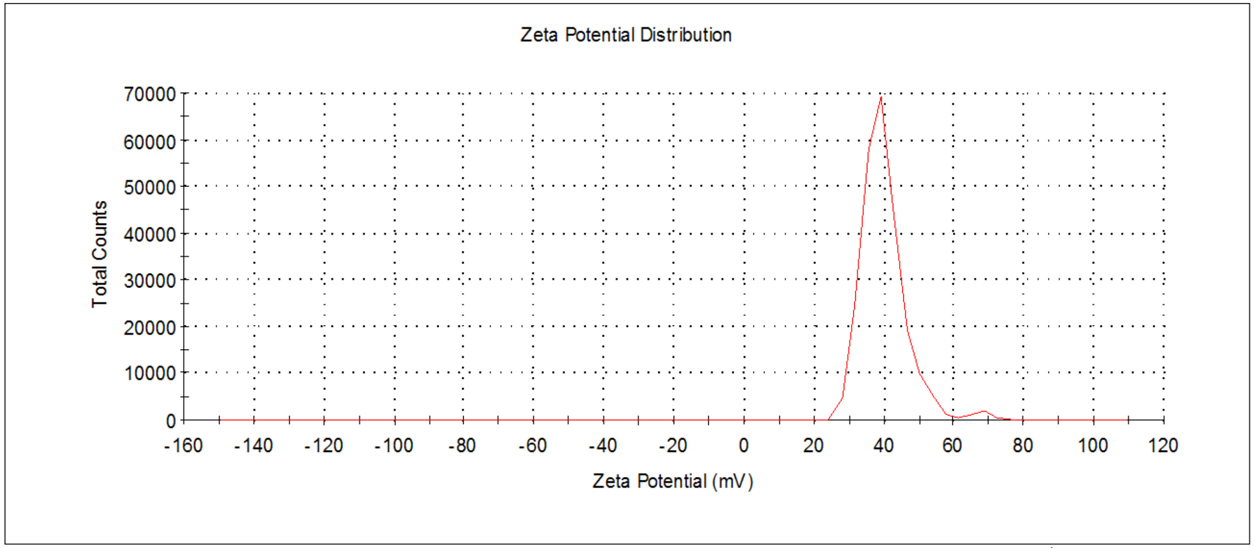

Figure S24: Zeta potential of solution of the macrocycle **11** in the water (1×10^{-4} M).

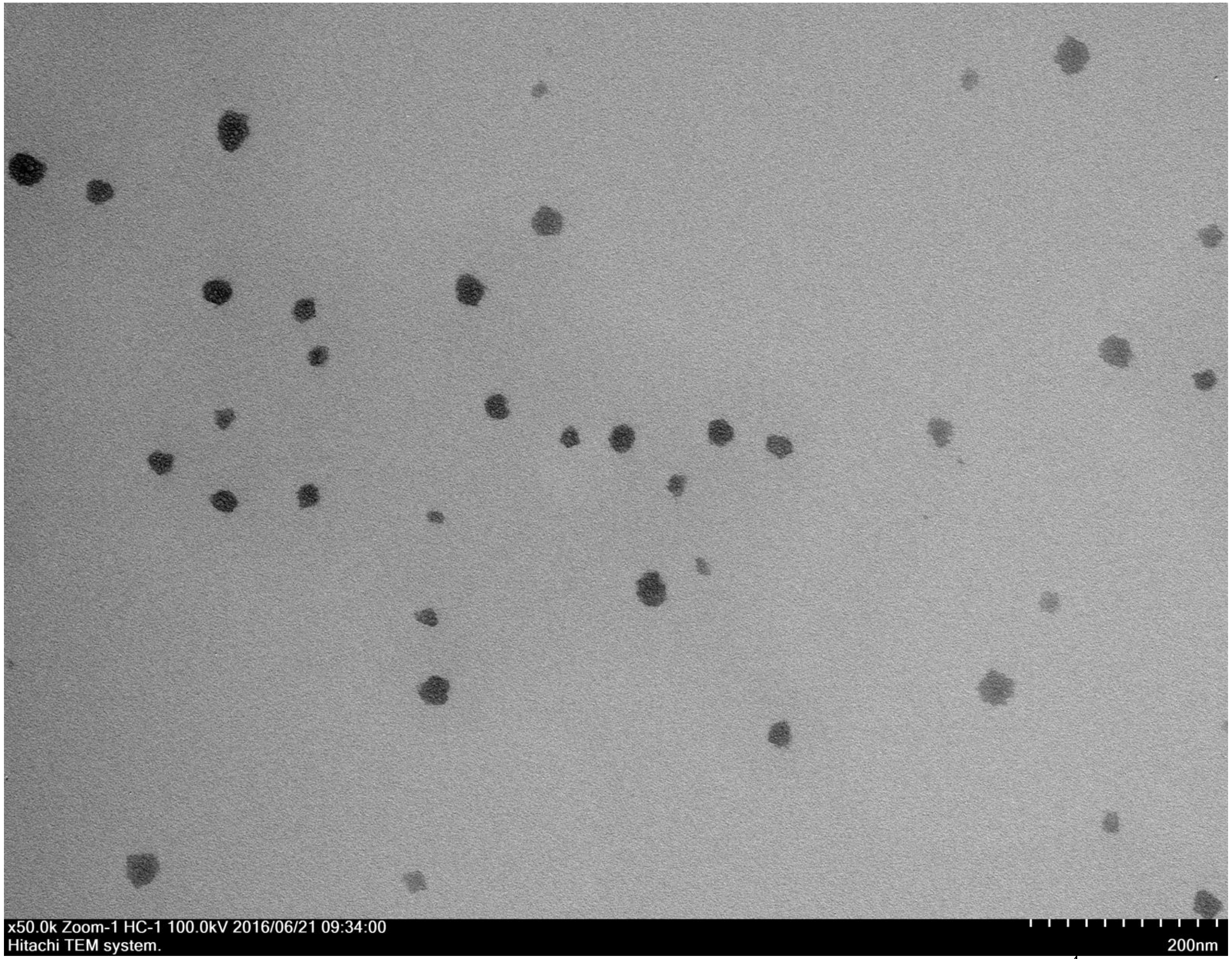

Figure S25: TEM image of self-associates based on thiacalix[4]arene *cone*-**8** in water (1×10^{-4} M). Scale bar 200 nm.

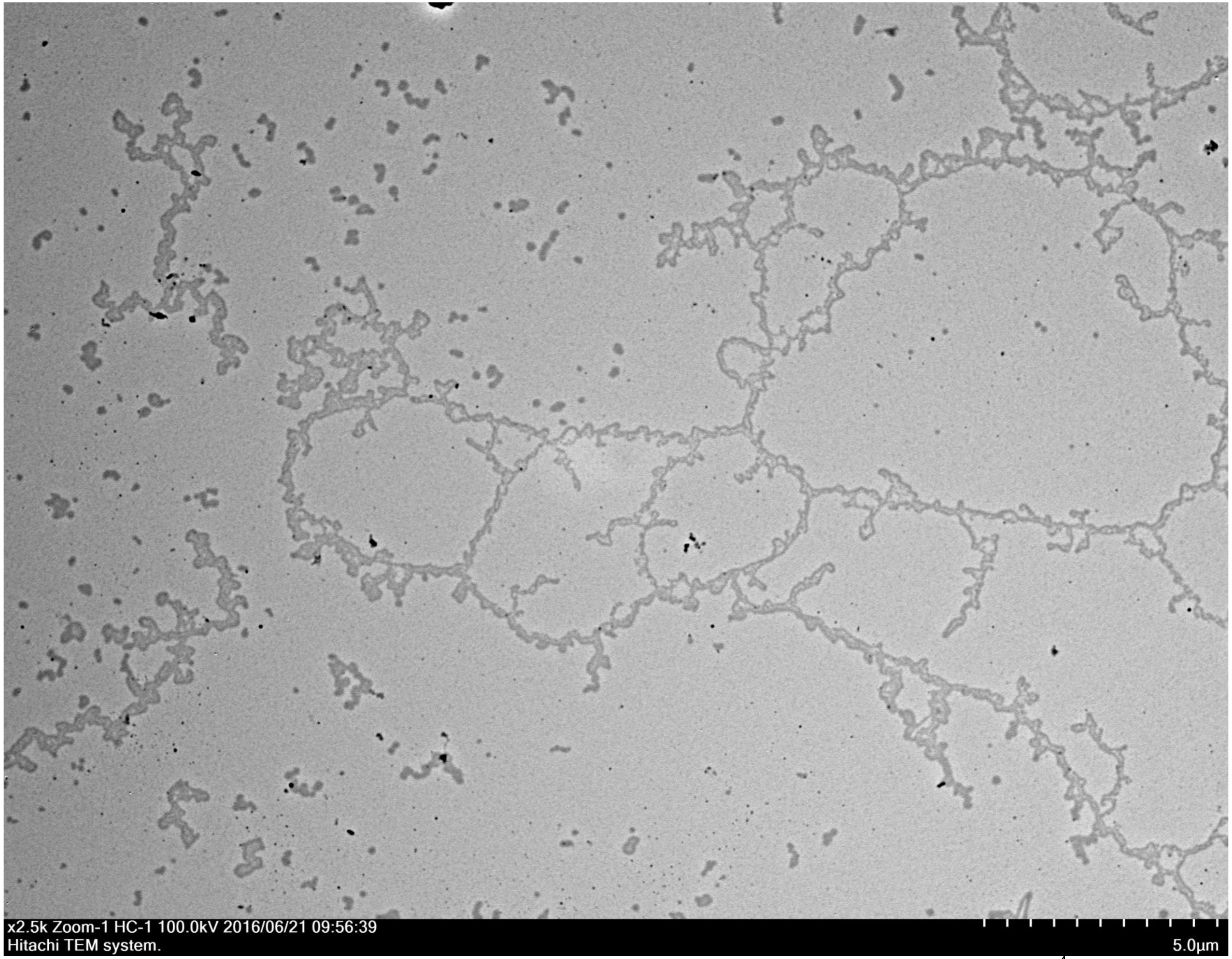

Figure S26: TEM image of self-associates based on thiacalix[4]arene 1,3-*alternate*-**9** in water (1 × 10^{-4} M). Scale bar 5 μm.

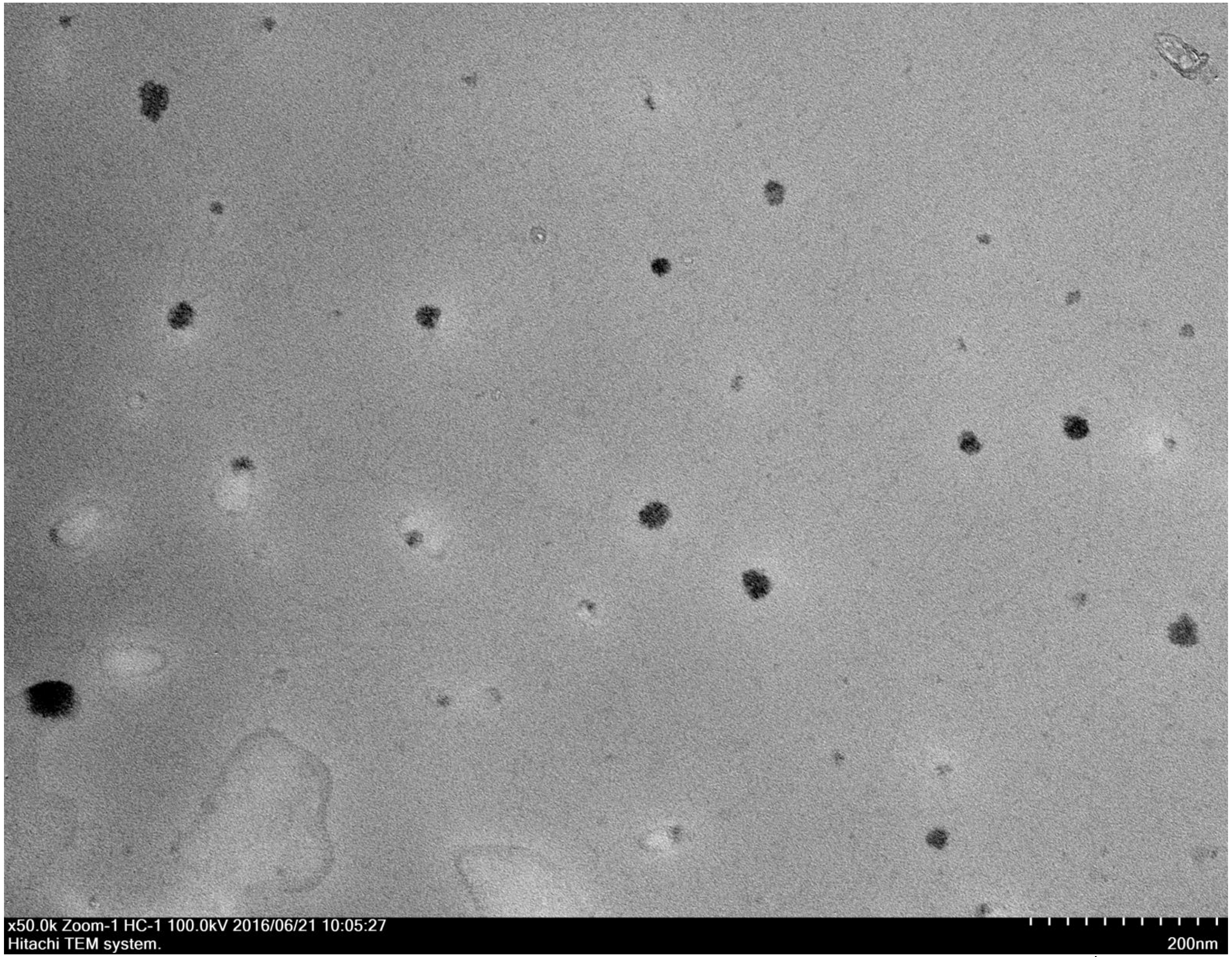

Figure S27: TEM image of self-associates based on thiacalix[4]arene *cone*-**10** in water (1×10^{-4} M). Scale bar 200 nm.

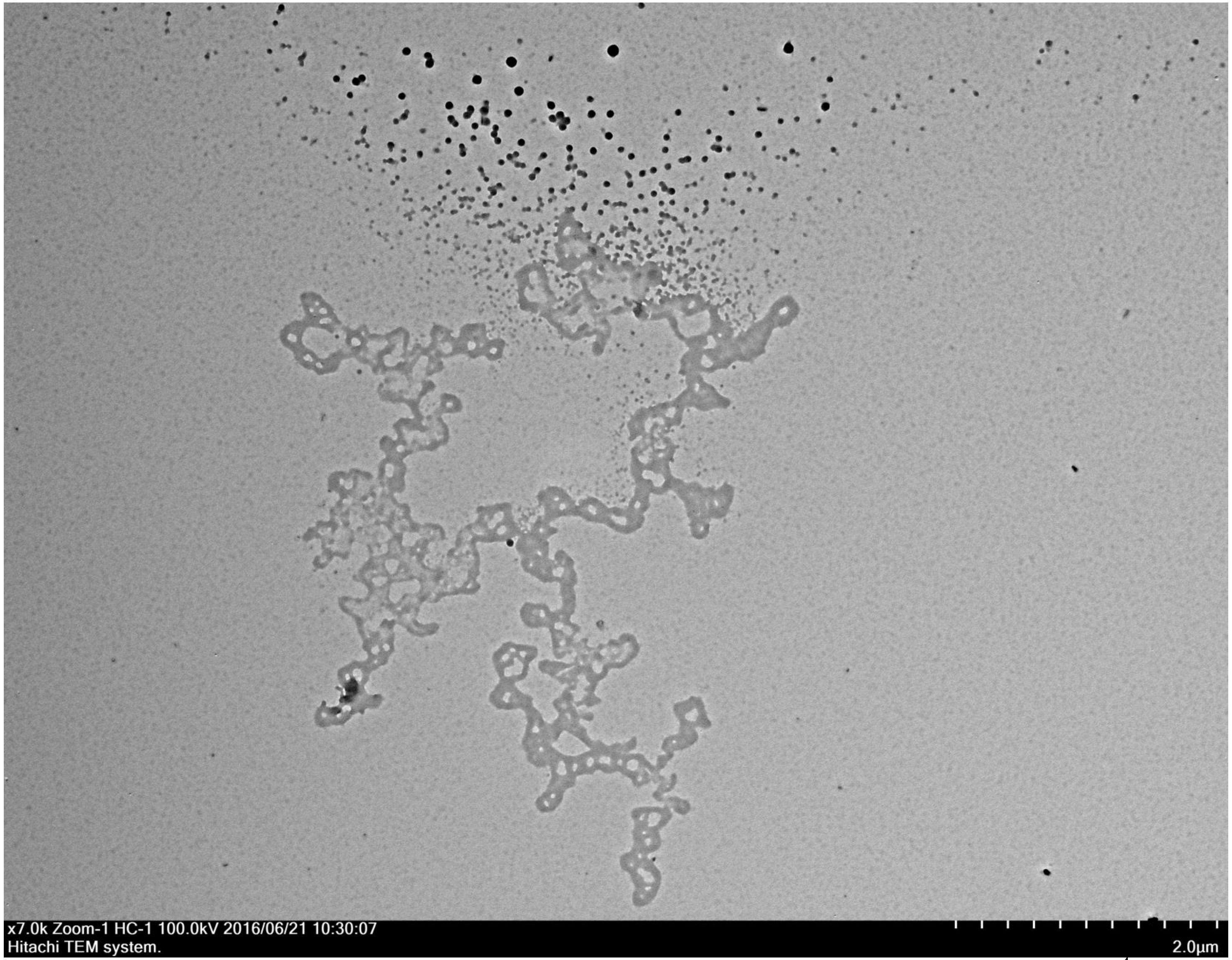

Figure S28: TEM image of self-associates based on thiacalix[4]arene 1,3-*alternate*-**11** in water (1×10^{-4} M). Scale bar 2 μm.

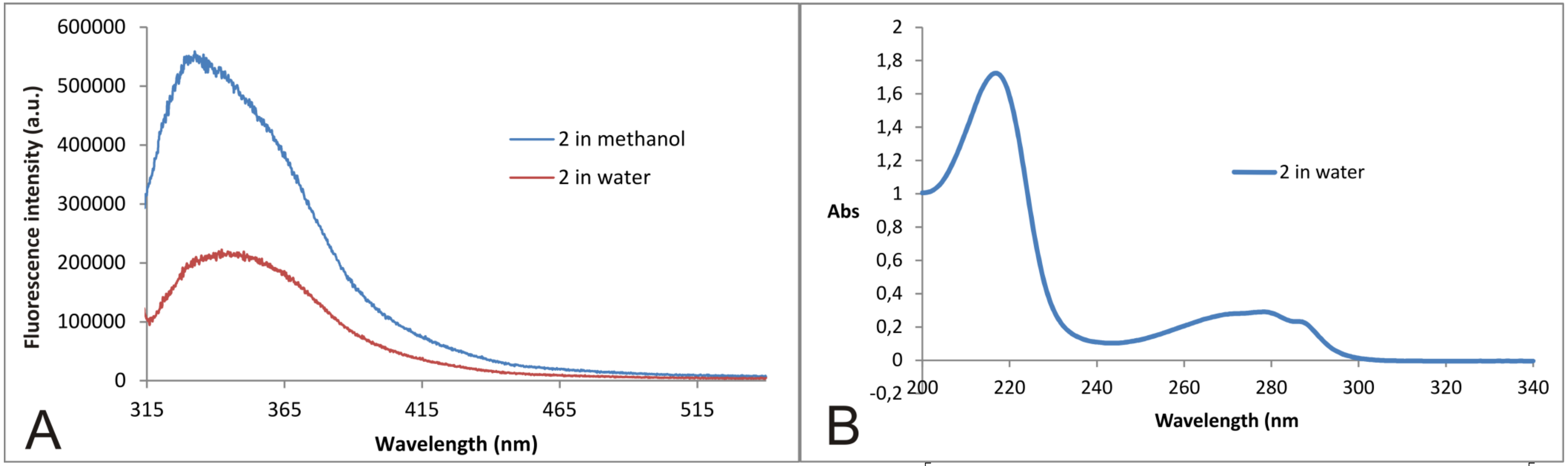

Figure S29: A) Fluorescence spectra of compound **2** in water and methanol (1×10^{-5} M), B) UV spectrum of compound **2** in water (1×10^{-5} M).

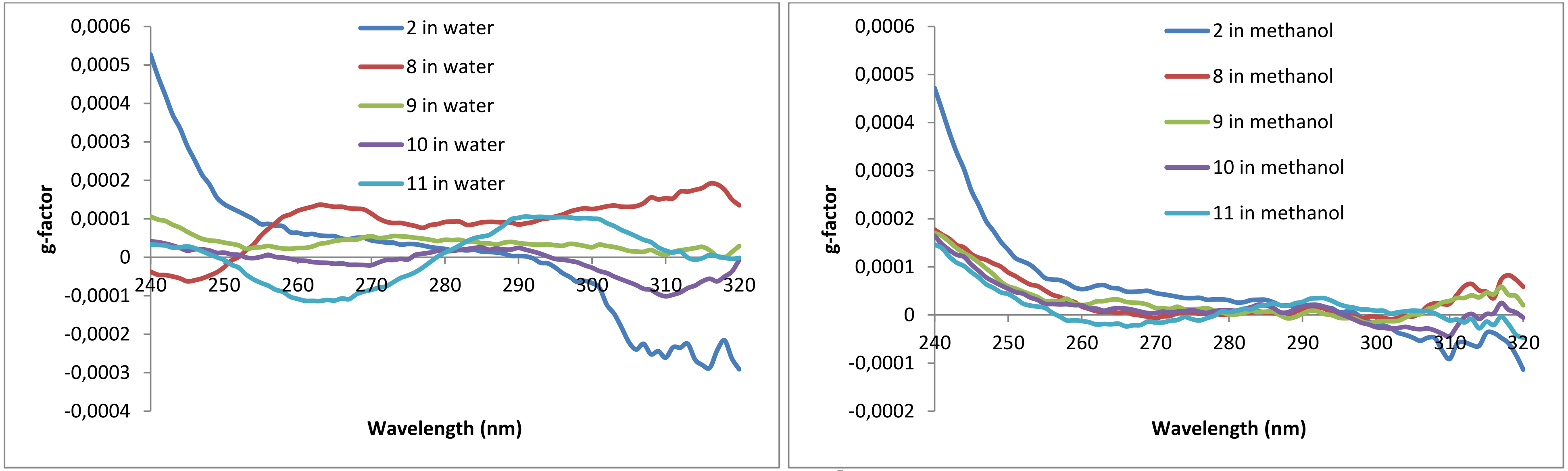

Figure S30: *g*-Factors of compounds **2** and **8–11** in water and methanol (1×10^{-5} M).

The optical activity of chiral systems is quantified using the anisotropy factor (*g*-factor) [1,2]:

$g = \Delta\varepsilon / \varepsilon$

where $\Delta\varepsilon$ and ε are the molar circular dichroism and molar extinction coefficient, respectively.

References

1. Yan, W.; Xu, L.; Xu, C.; Ma, W.; Kuang, H.; Wang, L.; Kotov, N. A. *J. Am. Chem. Soc.*, **2012**, *134*, 15114–15121.
2. Cheng, J.; Le Saux, G.; Gao, J.; Buffeteau, T.; Battie, Y.; Barois, P.; Ponsinet, V.; Delville, M.-H.; Ersen, O.; Pouget, E.; Oda, R. *ACS Nano*, **2017**, *11*, 3806–3818.
